# The anticancer potential of chemical constituents of *Moringa oleifera* targeting CDK-2 inhibition in estrogen receptor positive breast cancer using in-silico and in vitro approches

**DOI:** 10.1186/s12906-023-04198-z

**Published:** 2023-11-04

**Authors:** Rida Sultan, Abrar Ahmed, Li Wei, Hamid Saeed, Muhammad Islam, Muhammad Ishaq

**Affiliations:** 1 Faculty of Pharmacy, Punjab University College of Pharmacy, University of the Punjab, Lahore, 54590 Pakistan; 2 Zhongshan Institute for Drug Discovery, Shanghai Institute of Materia Medica, Chinese Academy of Sciences (CAS), Zhongshan, 528400 P. R. China; 3 Institute of Social and Cultural Studies, University of the Punjab, Lahore, 54590 Pakistan

**Keywords:** CDK2, ER^+^ Breast cancer, Molecular Docking, MM-GBSA, Molecular Dynamic Simulations, MTT assay, *Moringa oleifera*, Phytoconstituents, Chlorogenic acid

## Abstract

Most of the breast cancers are estrogen receptor-positive recurring with a steady rate of up to 20 years dysregulating the normal cell cycle. Dinaciclib is still in clinical trials and considered as a research drug against such cancers targeting CDK2.

The major goal of this study was to identify the potential inhibitors of CDK-2 present in *Moringa oleifera* for treating hormonal receptor positive breast cancers. For this purpose, in silico techniques; molecular docking, MM-GBSA and molecular dynamics simulations were employed to screen *Moringa oleifera* compounds and their anticancer potential was determined against CDK-2 protein targets. Among 36 compounds of *Moringa oleifera* reported in literature, chlorogenic acid (1), quercetin (2), ellagic acid (3), niazirin (4), and kaempferol (5) showed good affinity with the target. The interaction of the compounds was visualized using PYMOL software. The profiles of absorption, distribution, metabolism, excretion (ADME) and toxicity were determined using SWISS and ProTox II webservers. The MTT assay was performed in-vitro using MCF-7 cancer cell lines to validate the anticancer potential of *Moringa oleifera* leaf extract.

MTT assay results revealed no significant change in proliferation of Mcf-7 cells following 24 h treatment with fraction A (petroleum ether). However, significant antiproliferative effect was observed at 200 µg/mL dose of fraction B (ethyl acetate) and cell viability was reduced to 40%.

In conclusion, the data suggested that all the compounds with highest negative docking score than the reference could be the potential candidates for cyclin dependent kinase-2 (CDK-2) inhibition while ellagic acid, chlorogenic acid and quercetin being the most stable and potent inhibitors to treat estrogen receptor positive breast cancer targeting CDK-2. Moreover, the data suggested that further investigation is required to determine the optimum dose for significant antiproliferative effects using in-vivo models to validate our findings of *in-silico* analysis.

**Supplementary Information:**

The online version contains supplementary material available at 10.1186/s12906-023-04198-z.

## Introduction

Breast cancer being the most recurrent malignancy among women has a prevalence of above 1.3 million, attributing 23% of the carcinomas [1–3]. This cancer is of metastatic type spreading to other organs making it incurable [4]. Various screening approaches employed to detect breast cancer such as mammography or magnetic resonance imaging have reduced the mortality rate worldwide. The estrogen hormone released from ovaries is correlated with breast cancer risk in women [4]. Molecular subtypes of breast cancer include luminal A (ER + , PR + , -HER2), luminal B (ER + or PR + , + HER2), HER2 enriched (ER- or PR-, + HER2) and triple negative breast cancer (ER- or PR-, -HER2) [5, 6]. Most of these cancers are type A luminal ER positive with recurrence occurring at a constant rate till 20 years while for ER negative breast cancer recurrence rate is 3 to 5 years [7, 8]. Luminal A cancers grow more slowly, are of lower grade and have a better prognosis [9].

Dysregulation of the cell cycle is the hallmark of all types of cancer, and cell regulators are the potential targets for novel cancer therapeutics [10, 11]. Under normal circumstances, the cell cycle is composed of the following distinct phases in an order (G0, G1, S, G2 and M), and combinations of cyclin/CDK are vital in modulating the process [12, 13].

In mammalian cells two classes of cyclins involved during progression from G1 phase include: D-type (D1, D2, D3) and E-type (E1 and E2) [14, 15]. CDKs are serine/threonine protein kinases encoded by 12 separate genetic arrangements and this family of kinases include three interphase cyclin dependent kinases (CDK-2, CDK-4 and CDK-6), a mitotic (CDK-1), CDKs that regulate the processes (CDK-7) and transcriptional CDKs(CDK-8 and CDK-9) [16, 17]. Several studies verified modifications in regulators of cell cycle; such as cyclins, CDK and the retinoblastoma genes in human breast cancer [1, 18, 19].

The complex of CDK4/6 and Cyclin D early in G1 phase is associated with hypo-phosphorylation of retinoblastoma 1 gene preparing it for hyper-phosphorylation later in the G1 by CDK2 and cyclin E complex. It initiates the transcription factor release which are required for entry into next phase (S phase) of cell cycle [20]. The control of cell cycle in estrogen receptor positive patients is achieved by blocking the CDK4/6. Pan-CDK inhibitors developed initially caused severe toxicities and were proved unsuccessful in inhibiting cancer cells. However, the favorable tolerability by potent specific CDK inhibitors has developed the interest in the targeted therapies [7].

ER signaling is associated with upregulation of CDK4/6-Cyclin D complex promoting the tumor cell proliferation [1, 21]. So it has been established in recent years that endocrine therapy can combine with target cell therapy for ER-positive breast cancers [21]. 70% of the ER-positive patients are suitable candidates for this therapy [22]. ER down-regulators (Fulvestrant), ER-modulators (Tamoxifen) and aromatase inhibitors (AI's) combined with CDK4/6 have been the mainstay of treatment in advanced ER-positive patients for decades [21, 22].

Palbociclib, ribociclib and abemaciclib are potent inhibitors of CDK-4 and 6 and bind to the ATP left and are approved by Food and Drug administration of United States for use in breast cancer [7, 23]. Dinaciclib that inhibits the CDK2 is currently in clinical trials for breast cancer [24–26]. Despite various advanced treatment tools, resistance developed in therapies that are often fatal require new treatment approaches [1].

phytoconstituents and their derivatives have been less toxic and are better option for cancer treatment based on research [27, 28]. Various primary and secondary metabolites play an important role in inhibiting CDKs and signaling pathways [29]. Kaempferol (a flavanol) derived from plants inhibits the activity of several enzymes i.e., CDK2 and CDK4 and cell cycle arrest at following phases G1, G2 and M phase [28, 30]. Several other flavonoids and alkaloids from plants are derived as anticancer agent [31].

*Moringa oleifera*, a marvelous tree, belonging to family Moringaceae exhibits many pharmacological activities. It is used in many bacterial and fungal infections. It is a powerful cardio-tonic and has an anti-oxidant, anti-cancer, anti-epileptic and anti-inflammatory properties as well. It is used to lower the glucose levels in diabetic patients and blood pressure in others. It is also used to treat ulcers [32–34]. All of these activities are attributed to its constituents including alkaloids (moringine, moringinine), nitrile glycosides such as niazirin, glycosides of mustard oil, niaziminin A and B, flavonoids (kaempferol, quercetin, isoquercetin, rutin), phenolic acids (such as chlorogenic acid, gallic acid and ellagic acid), Vitamins and β-carotenes, essential amino acids (such as methionine, cystine and lysine), vanillin, 4-hydroxymellein, β-sitosterol and octacosanoic acid, aurantiamide acetate, 1,3 dibenzyl-urea [32, 35, 36].

Herein, we report the anticancer potential of phytoconstituents from *Moringa oleifera* using sophisticated *in-silico* strategies targetting CDK2. The binding interactions of the ligands were determined by molecular docking, prime MM-GBSA energy calculations as well as molecular dynamic MD simulations. Michigan Cancer Foundation-7 (Mcf-7) breast cancer cell line was used for in vitro studies to observe the anticancer potential of *Moringa oleifera* extract.

## Materials and methods

### *In-silico* analysis

#### Protein preparation

The X-ray crystal structure of CDK-2 (Protein Data Bank ID: 2XMY) was fetched from PDB. Protein preparation Wizard located in Maestro v13.2 Schrödinger, LLC, 2022.2 (https://www.schrodinger.com/products/maestro) software package was used to prepare the protein. The missing residues were added and crystallographic water molecules were removed. H-Bonds were adjusted at variable pH and bond orders were assigned. Finally, structures were protonated and minimized using Optimized Potentials for Liquid Simulations force field at pH (7.0).

#### Ligand preparation

The structures of 36 phytoconstituents reported in the literature from *Moringa oleifera*:1,3 dibenzyl urea, moringine, moringinine, niazirin, niazirinin, niaziminin, niazimicin, benzyl isothiocyanate, acids like (gallic, chlorogenic, ellagic, ferulic), kaempferol, quercetin and isoquercetin, rutin, lutein, beta carotene, vitamin B2, nicotinic acid, pyridoxine, folic acid, tocopherol, ascorbic acid, methionine, cystine, tryptophan, lysine, β-sitosterol, stigmasterol, octacosanoic acid,4-hydroxymellein,aurantiamide acetate, 4-[(4'-O-acetyl-α-L-rhamnosyloxy) benzyl] isothiocyanate, o-ethyl-4-[(α-L-rhamnosyloxy)-benzyl] carbamate, vanillin and dinaciclib as a reference drug for breast cancer were drawn on 2D sketch program available in Maestro v13.2 Schrödinger, LLC, 2022.2 software package. All ligands were prepared using Lig-Prep module available in Maestro v13.2, Schrödinger, LLC, 2022.2. The energy minimization was done by OPLS (2005) force field.

#### Molecular docking studies

Molecular docking studies were performed using Glide with default parameters available in Maestro 13.2 (Glide, Schrödinger, LLC, 2022.2). First, a binding pocket was located using receptor grid generation constituting the key residues involved in ligand binding. Molecular docking (XP) calculations were performed using Glide at the binding site of CDK2 protein with default parameters. No constraints were applied for all the docking studies. For each compound, multiple poses were attained after the molecular docking calculations containing the key residues involved in ligand binding.

#### Prime/MM-GBSA simulation

Prime MM-GBSA method was used to calculate the binding free energy (**∆**G-bind) of ligands based on docking complex by following equation:

$$\Delta\mathbf{Gbind}\boldsymbol\;=\;\mathrm\Delta\mathbf{EMM}\boldsymbol\;+\;\mathrm\Delta\mathbf{Gsolv}\boldsymbol\;+\;\mathrm\Delta\mathbf{GSA}$$

Where ∆EMM is the difference in energy minimized between CDK2 and inhibitor complex, ∆Gsolv is difference in solvation energy GBSA of CDK2 and inhibitor complex, ∆GSA is difference in energies of surface area for CDK2 and inhibitor complex.

OPLS force field and GB/SA continuum solvent model was used to calculate energies of the complexes [37, 38].

#### Molecular dynamic simulations

Maestro-Desmond v12.3 Schrödinger software package was used to evaluate stability and interaction of CDK-2 receptor protein with suitable ligands. The water molecules were placed with the docking complex and system was neutralized. At normal temperature and pressure MD simulations were run for 100 ns. Root mean square fluctuation (RMSF) of ligands and Root mean square deviation (RMSD) of complex and amino acid residues involved in contacts were observed.

#### Pharmacokinetic analysis

The compounds were studied using Swiss ADME webserver which provided their ADME (absorption, distribution, metabolism and excretion) profiles predicting their pharmacokinetics. The data obtained included number of rotatable bonds, H-bond acceptors, H-bond donors, log values P_o/w_ and GI absorption.

#### Pharmacodynamic analysis

All were subjected to pharmacodynamic studies using the online webserver ProTox-II which determined the toxicity profiles of the compounds. The data calculated includes LD50 mg/kg and toxicity class of the compounds.

### *In-vitro* studies

#### Plant material

The leaves of *Moringa oleifera* were collected in December from bachelor male teacher hostel, University of the Punjab, Lahore, following proper guidelines and legislation procedures. The plant was shade dried. The plant was authenticated by an expert taxonomist from the Department of Botany, Government University (GCU), Lahore Pakistan. A voucher specimen was deposited under the reference number GC. Herb. Bot. 3870.

#### Solvents and chemicals

Ethanol (95%) (BDH laboratory-England), Deionized water, Petroleum ether (BDH laboratory-England), Ethyl acetate (BDH laboratory-England), Rotary evaporator (Heidolph-Germany), Separating funnel. Dulbecco’s modified eagle medium (DMEM) (caisson labs, USA), fetal bovine serum (FBS) (Hyclone, South America) penicillin–streptomycin (Hyclone, US), Phosphate buffered saline (PBS) (Oxoid, England), (3-(4, 5-dimethylthiazol-2-yl)-2, 5-diphenyltetrazolium bromide) (MTT) (Bioworld, USA), Dimethyl sulfoxide (DMSO), syringe filters 0.2 µm, Eppendorf tubes, T-flask 25 cm^2^ (corning, USA), hemocytometer, 96 well plates (corning, USA), petroleum ether dilutions (100 µg/mL, 200 µg/mL and 300 µg/mL), ethyl acetate dilutions (100 µg/mL, 200 µg/mL and 300 µg/mL).

#### Preparation of extracts and fractions

After drying cold extraction was carried out by macerating 1000 g of powdered leaves using ethanol as a solvent. The extract was evaporated. The extract was concentrated by evaporating solvent using rotary evaporator at 45–50°c and repeated the procedure three times. The concentrated crude extract was sequentially fractioned with petroleum ether and ethyl acetate to obtain Fr. A and Fr. B respectively. These two fractions were freeze dried in petri dish and stored for use in lab work.

#### Cell culture

Michigan Cancer Foundation-7 (Mcf-7) cell line was provided by Dr. Azra from School of Biological Sciences (Centre of Excellence in Molecular Biology, CEMB), PU, Lahore.

The cells were maintained in DMEM supplement along with 10% FBS (v/v) and 1% penicillin/streptomycin solution (v/v) at 37 °C temperature, 5% CO2 and 95% relative humidity.

#### MTT assay

The anticancer potential of *Moringa oleifera* leaves extract was evaluated as described by Karakaş, Ari, & Ulukaya with slight modifications at University College of pharmacy, University of the Punjab, Lahore during the year 2022. Briefly, the cells were plated in a 96-well plate at a density of 2500 cells per cm^2^, incubated for 24 h under 5% CO2 at 37 °C temperature, followed by treatment with different concentrations (100 µg/mL, 200 µg/mL and 300 µg/mL) of fractions along with 150 µL of DMEM media. After 24 h, 10 µL of MTT reagent (5 mg per mL in DMSO) was added in each well and subjected to further incubation for 3 h after which 80% medium was flicked off and formazan crystals formed were solubilized in 150 µL of DMSO, incubated for half an hour. The absorbance was then measured using ELISA reader at 500–600 nm. The percentage of viable cells was calculated using the following formula:

$$\mathrm{\%\,Cell\,viability}= 100 \times \frac{sample\left(abs\right)-blank(abs)}{sample\left(abs\right)-blank(abs)}$$

#### Statistical analysis

GraphPad Prism 8 is used for statistical analyses. The cell viability experiments were performed in triplicate and the results was expressed as mean ± standard error the mean (SEM). Statistical significance was calculated using one way analysis of variance (ANOVA). A value of *P* < 0.05 was taken as statistically significant.

## Results

### *In-silico* results

#### Molecular docking

**Fig. 1:**
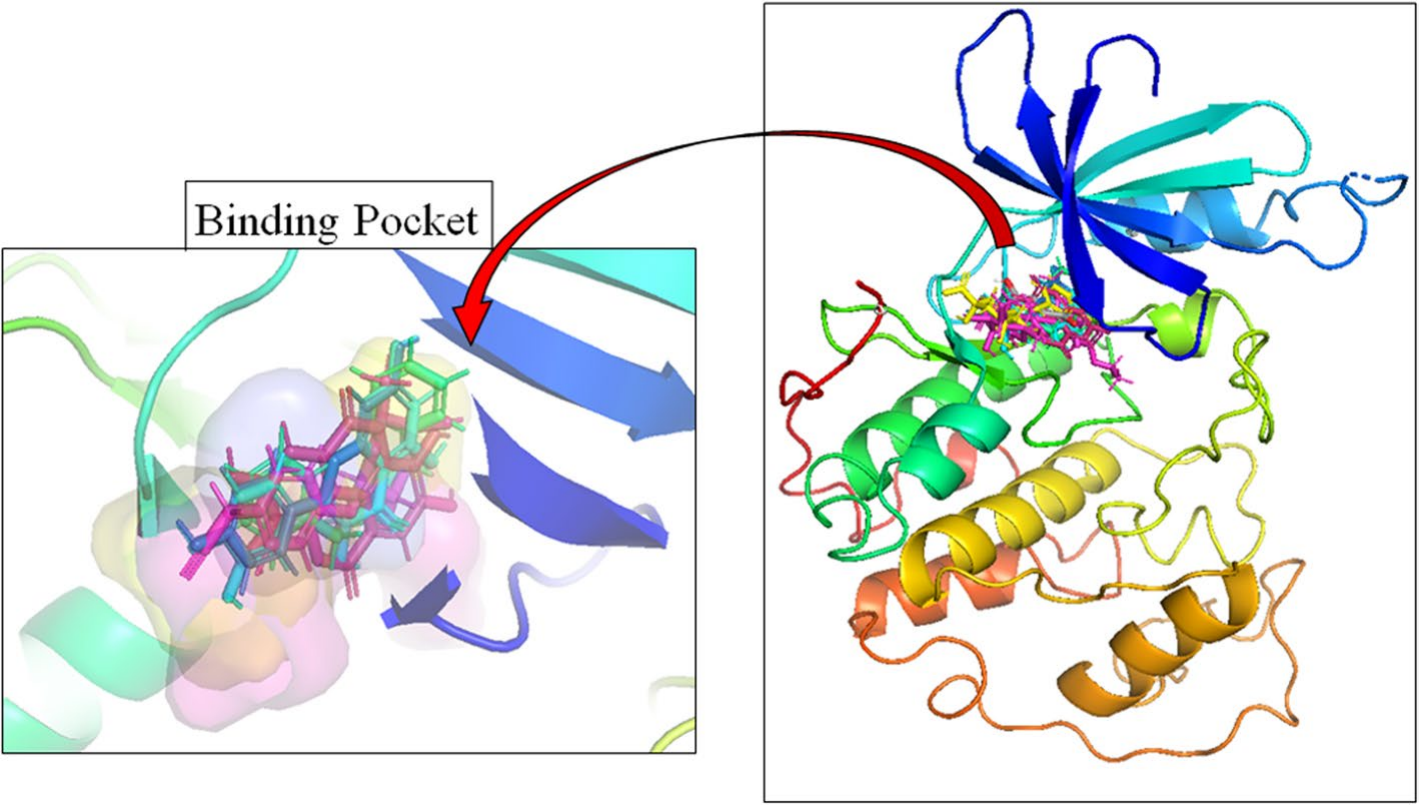
The structure of CDK-2 enzyme (PDB: 2XMY) and docked ligands sharing same binding pocket

**Fig. 2:**
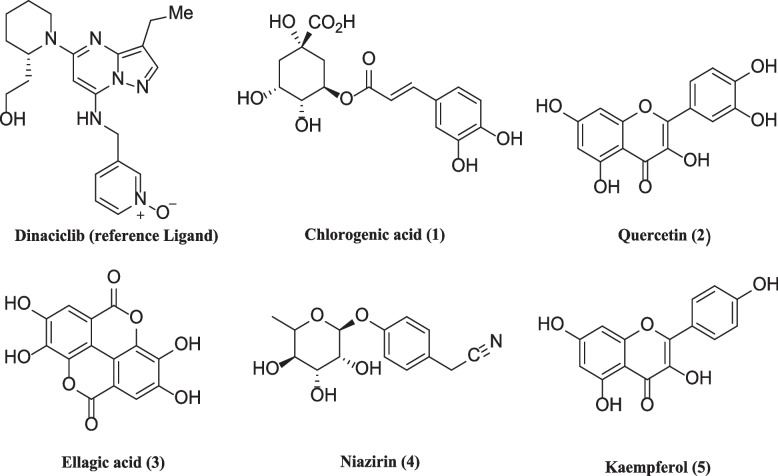
Two dimensional structures of Dinaciclib (reference ligand) and phytoconstituents from *Moringa oleifera*

The 36 phytoconstituents extracted from *Moringa oleifera* reported in literature along with dinaciclib (reference ligand) were docked with CDK-2 and were ranked in order of their affinities towards the protein (CDK-2) (Fig. 1). A potent CDK-2 inhibitor dinaciclib was used as a reference ligand. The ligands with potency greater than the dinaciclib were selected for further analysis as shown in Fig. 2.

Overall, only Chlorogenic acid (1), quercetin (2), ellagic acid (3), niazirin (4) and kaempferol (5), out of 36 screened ligands showed strong interactions with the CDK-2 protein with good docking score as shown in Table 1. All ligands including dinaciclib exhibited both polar and nonpolar, hydrogen and hydrophobic interactions, pi-pi stacking, salt bridges as well as cation-π contacts with the target protein residues.

**Table 1:** Molecular docking results and interaction of *Moringa oleifera* compounds with CDK2 protein

Ligand	Name	Binding affinity	Glide Energy	XP- H bond	Glide-ligand efficiency
Reference	Dinaciclib	-5.445	-43.761	-0.785	-0.188
1	Chlorogenic acid	-9.119	-53.061	-3.335	-0.365
2	Quercetin	-7.501	-47.010	-1.830	-0.339
3	Ellagic acid	-7.138	-45.633	-1.920	-0.321
4	Niazirin	-6.835	-43.100	-1.938	-0.342
5	Kaempferol	-6.717	-43.901	-1.482	-0.318

The affinities of ligands ranged from -6,717 to -9.119 kcal/mol. Chlorogenic acid (1), quercetin (2), ellagic acid (3), niazirin (4), kaempferol (5) had affinities -9.119, -7.501, -7.138, -6.835 and -6.717 kcal per mol respectively and greater than the the binding affinity of dinaciclib -5.445 kcal/mol. However, glide ligand efficiency which is percentage/potency efficiency index (PEI) of these compounds ranges from -0.318 to -0.365 and it is higher than -0.188, efficiency of dinaciclib. The efficiency refers to the ability of the compounds to produce desired clinical effects, which could be beneficial during optimization. The ligand efficiency determines the optimal interactions between the ligand and the receptor. It is a ratio of Gibb free energy (∆G) to non-hydrogen atoms of compound [39]. It measures the binding energy of each atom of ligand bound to receptor or enzyme. The difference in binding affinities is because of different functional groups like side chains and hydroxyl groups in the structure of the ligands. In molecular docking analysis ligands were ranked on the basis of their best binding poses and higher affinity values in negative and were further analyzed to determine their stability in the protein binding pocket.

#### Hydrogen bonding and hydrophobic interactions

Hydrogen bonds and hydrophobic interactions were visualized using PyMOL-2.5.2 software. The dinaciclib reference ligand (a potent CDK2 inhibitor) formed two conventional hydrogen bonds and 16 hydrophobic interactions. Chlorogenic acid (1) showed five conventional and one aromatic H-bond along with 21 hydrophobic interactions. Quercetin (2) exhibited two conventional and one aromatic H-bond and 19 hydrophobic interactions. Ellagic acid (3) showed four conventional and one aromatic H-bond as well as 19 hydrophobic interactions while niazirin (4) showed four conventional hydrogen bonds together with 19 hydrophobic interactions. Kaempferol (5) showed two conventional and three aromatic H-bond as well as 19 hydrophobic interactions. No aromatic H-bonds were observed in niazirin; however, it showed pi-pi stacking between two residues Phe-4 and Tyr-77 as shown in Table 2

**Table 2:** Hydrogen bond parameters derived from molecular docking of CDK-2 protein with compounds

Ligand	Name	Hydrogen Bonding	Distance Å
**Reference**	Dinaciclib	Gln131:H—Lig: N	2.5
		Lys9:H—Lig: O	2.0
**1**	Chlorogenic acid	Asp145:O—Lig: H	2.1
		His84:O—Lig: H	1.7
		Lys89:H—Lig: O	2.4
		Lys89:H—Lig: O	2.1
		Ile10:O—Lig: H	2.1
		Phe82:C—Lig: O	Aromatic
**2**	Quercetin	Asp145:O—Lig: H	1.9
		His84:O—Lig: H	1.8
		Asp86:O—Lig: H	Aromatic
**3**	Ellagic acid	Asp145:O—Lig: H	1.9
		Asp145:O—Lig: H	1.9
		Asp86:O—Lig: H	2.6
		Leu83:O—Lig: H	1.9
		Leu83:O—Lig: H	Aromatic
**4**	Niazirin	Asp145:O—Lig: H	1.9
		Asn132:O—Lig: H	1.8
		Gln131:O—Lig: H	2.1
		Lys89:H—Lig: O	2.4
**5**	Kaempferol	Asp145:O—Lig: H	1.9
		His84:O—Lig: H	1.7
		Asp145:O—Lig: H	Aromatic
		Ile10:O—Lig: H	Aromatic
		Leu83:O—Lig: H	Aromatic

#### Polar and non-polar interactions of ligands

**Fig. 3:**
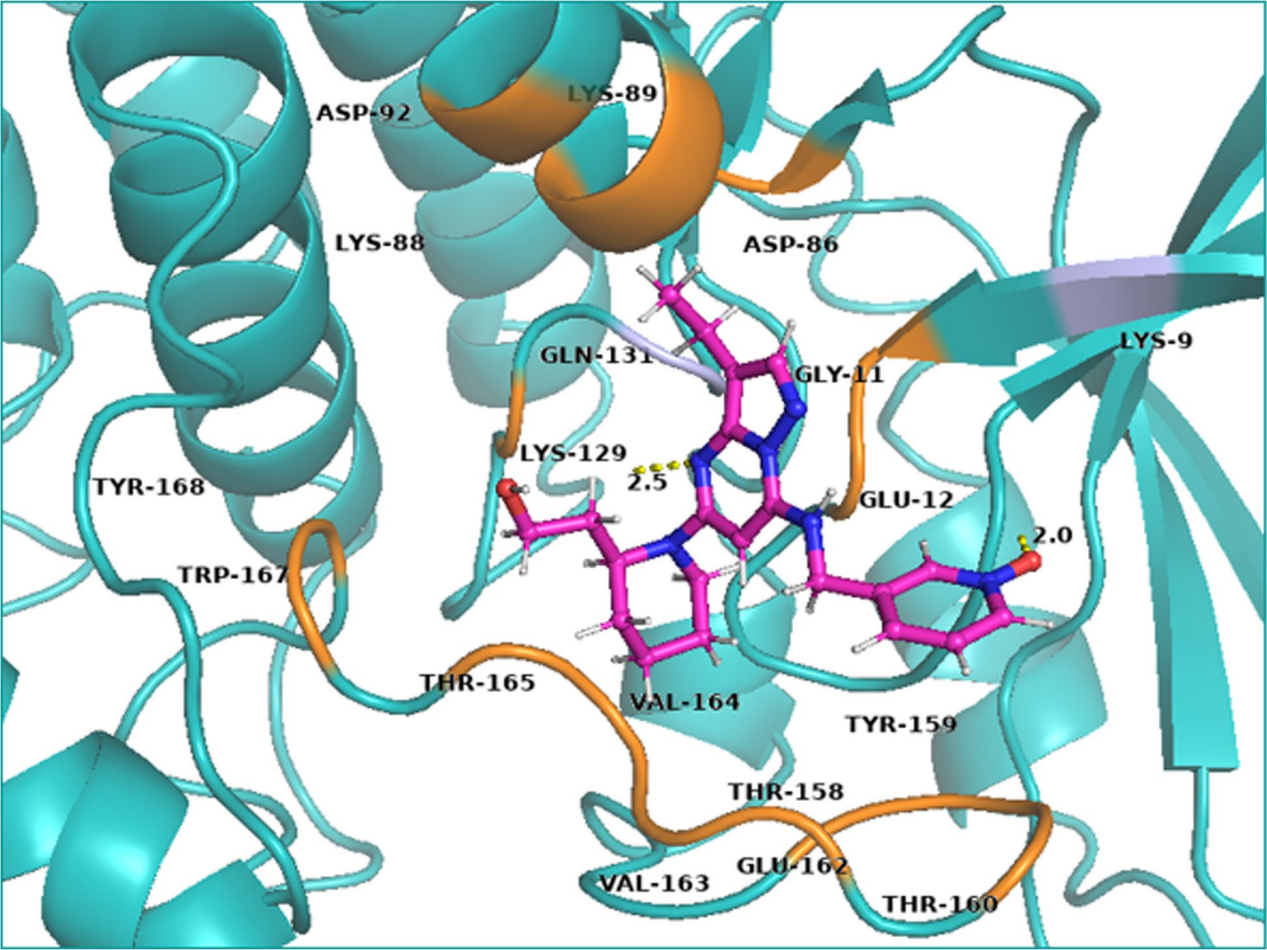
3D presentation of Dinaciclib (reference ligand) in the active site of CDK-2 protein (PDB code: 2XMY)

**Fig. 4:**
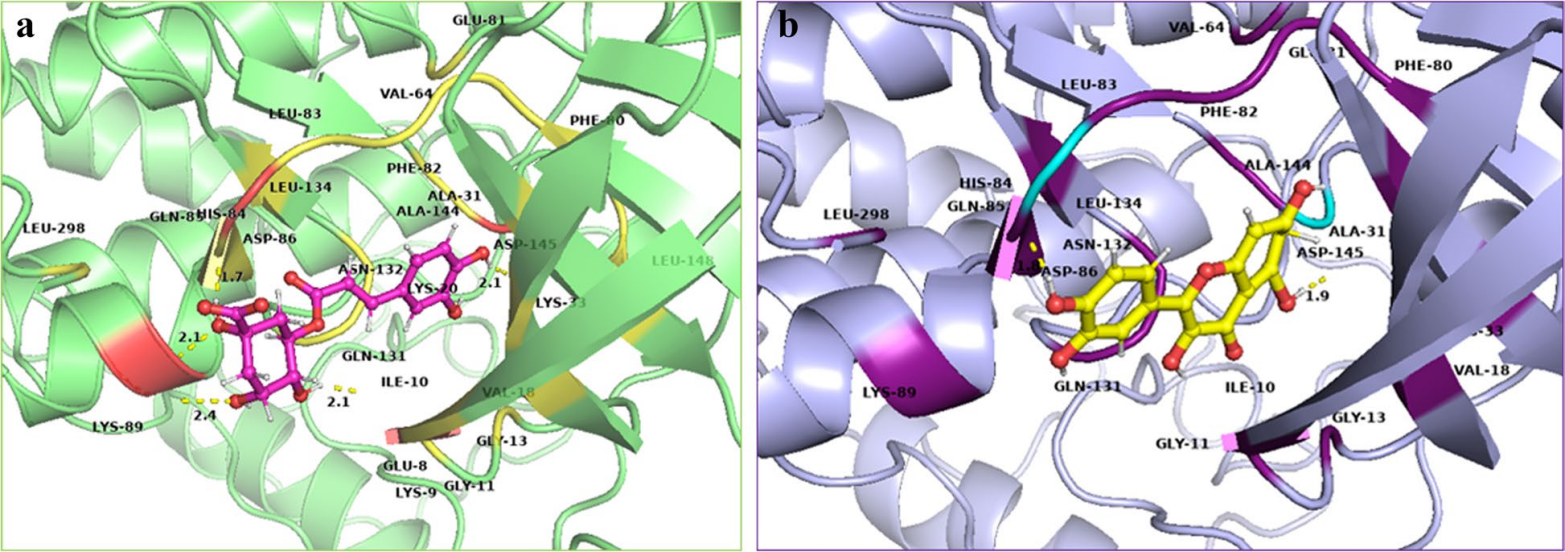
3D presentation of top ranked compounds **a** Chlorogenic acid [1] (pink), **b** Quercetin [2] (yellow), in the active site of CDK-2 protein (PDB code: 2XMY)

**Fig. 5:**
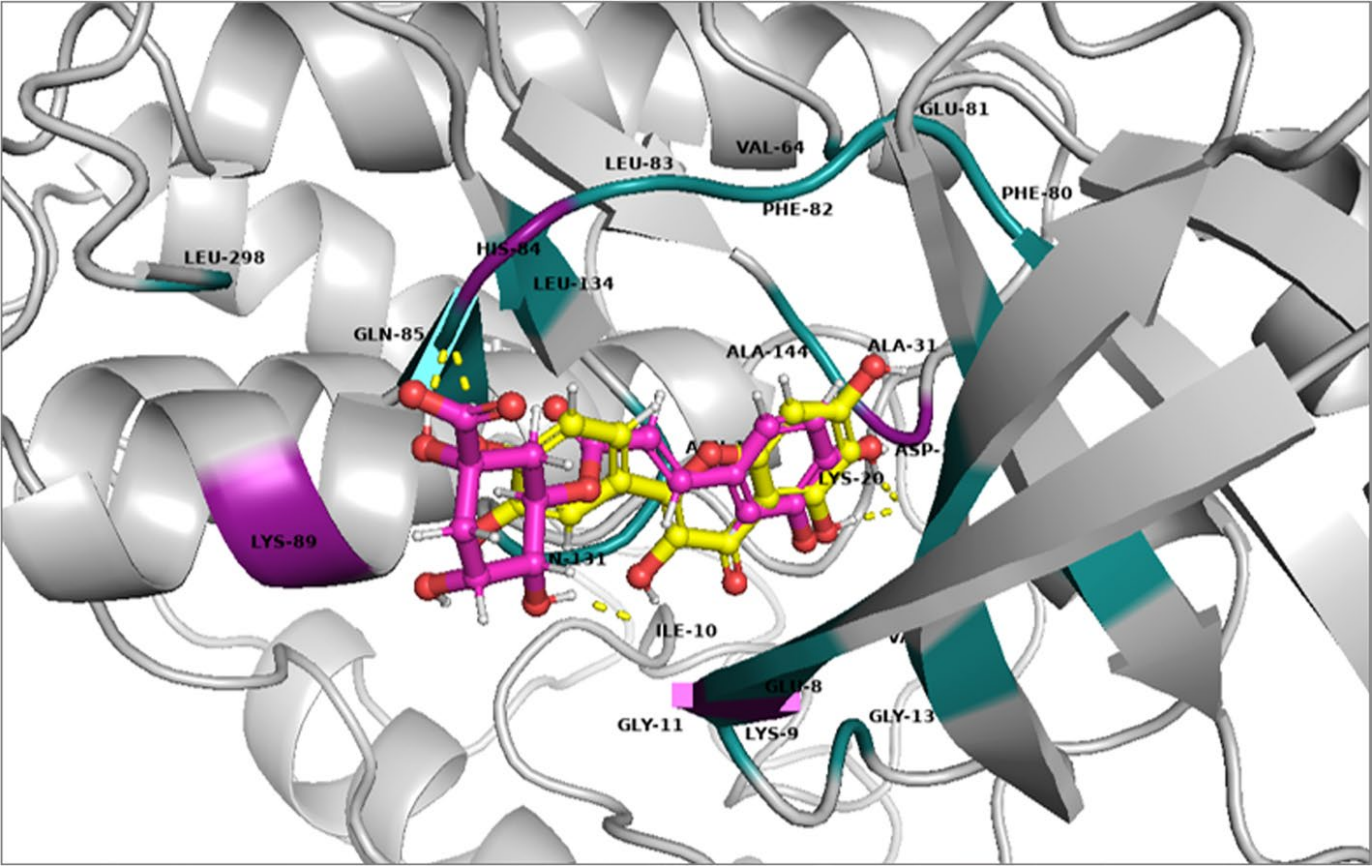
3D presentation of top ranked compounds; Chlorogenic acid [1] (pink) and Quercetin [2] (yellow), overlapped in the active site of CDK-2 protein, PDB code: 2XMY; polar residues in purple and nonpolar in deep teal

The polar and non-polar interactions were visualized using PyMOL-2.5.2 and key amino acid residues were identified. Ala-31, Ala-144, Gln-85, Gly-11, Gly-13, Lys-33, Leu-134, Phe-80, Phe-82, Val-18 and Val-64 showed non-polar contacts with all ligands while Glu-81 showed non-polar contacts with all ligands except niazirin [4]. Asp-145 showed polar contacts with all the ligands. The reference ligand Dinaciclib showed polar interactions with Gln-131 and Lys-9 while non-polar contacts with Asp-86, Asp-92, Glu-12, Glu-162, Gly-11, Thr-158, Thr-160, Thr-165, Tyr-159, Tyr-168, Trp-167, Lys-88, Lys-89, Lys-129, Val-163 and Val-164 as shown in Fig. 3. Chlorogenic acid (1) showed polar interactions with Asp-145, His-84, Lys-89 and Ile-10 while non-polar contacts with Asn-132, Asp-86, Glu-8, Lys-9, Lys-20, Leu-148 and Leu-298 as shown in Fig. 4a. Quercetin (2) showed polar contacts with Asp-145 and His-84 while non-polar contacts with Asn-132, Asp-86, Gln-131, Ile-10, Leu-83, Lys-89 and Leu-298 as shown in Fig. 4b. Chlorogenic acid (1) and Quercetin (2) are overlapped in the active site of CDK-2 protein as shown in Fig. 5.

**Fig. 6:**
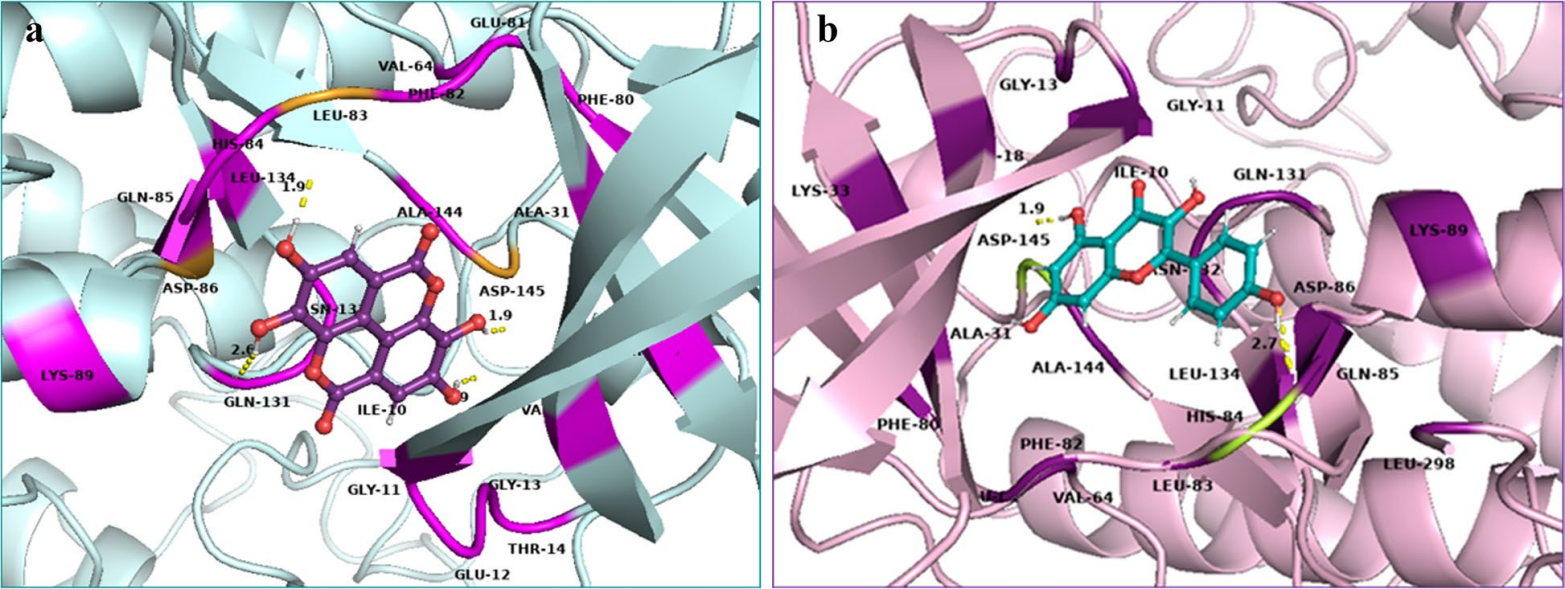
3D presentation of top ranked compounds **a** ellagic acid [3] (deep purple), **b** Kaempferol [5] (deep teal), in the active site of CDK-2 protein (PDB code: 2XMY)

**Fig. 7:**
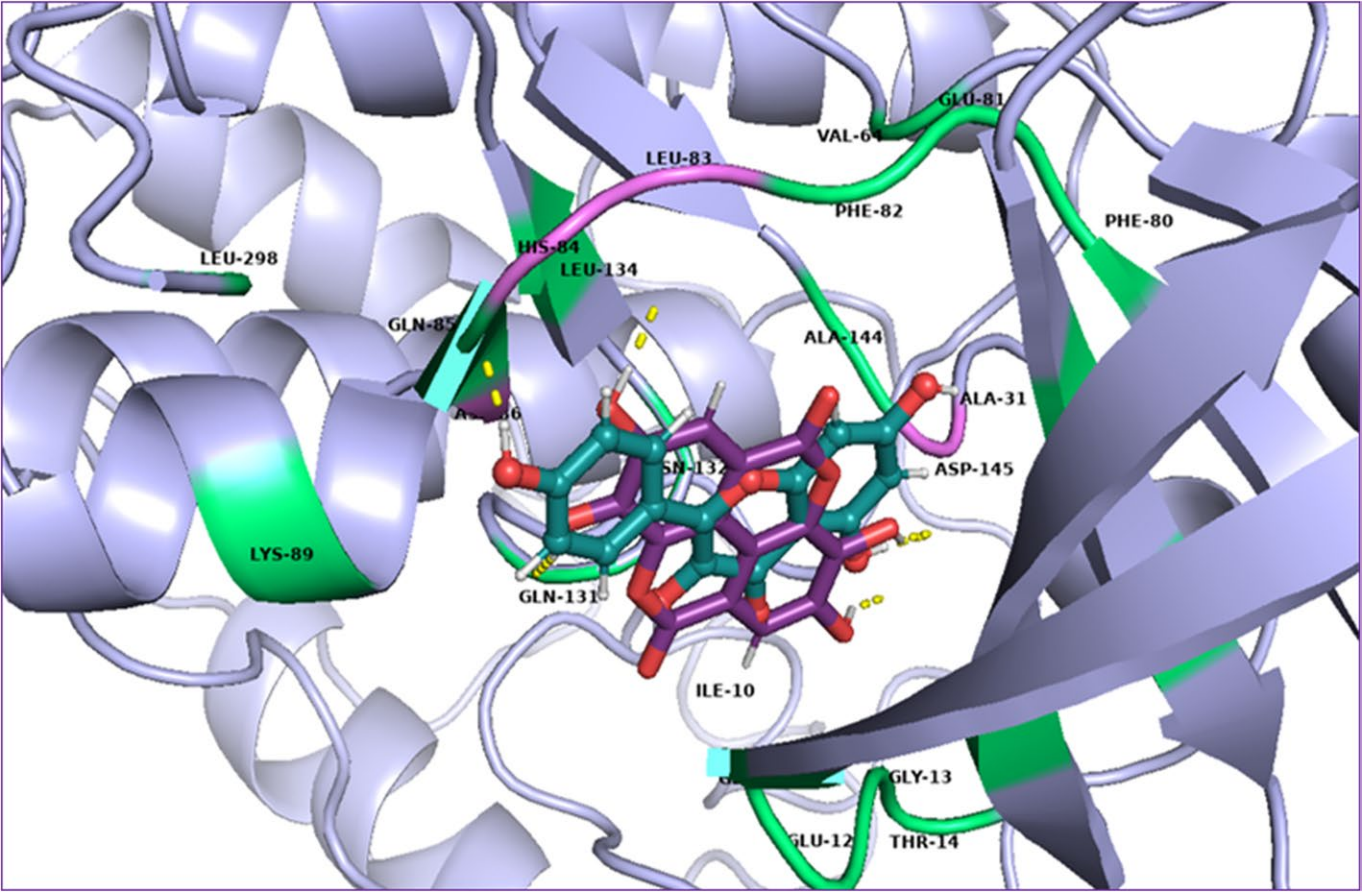
3D presentation of top ranked compounds; Ellagic acid [3] (deep purple) and Kaempferol [5] (deep teal), overlapped in the active site of CDK-2 protein, PDB code: 2XMY; polar residues in magenta and nonpolar in lime green

**Fig. 8:**
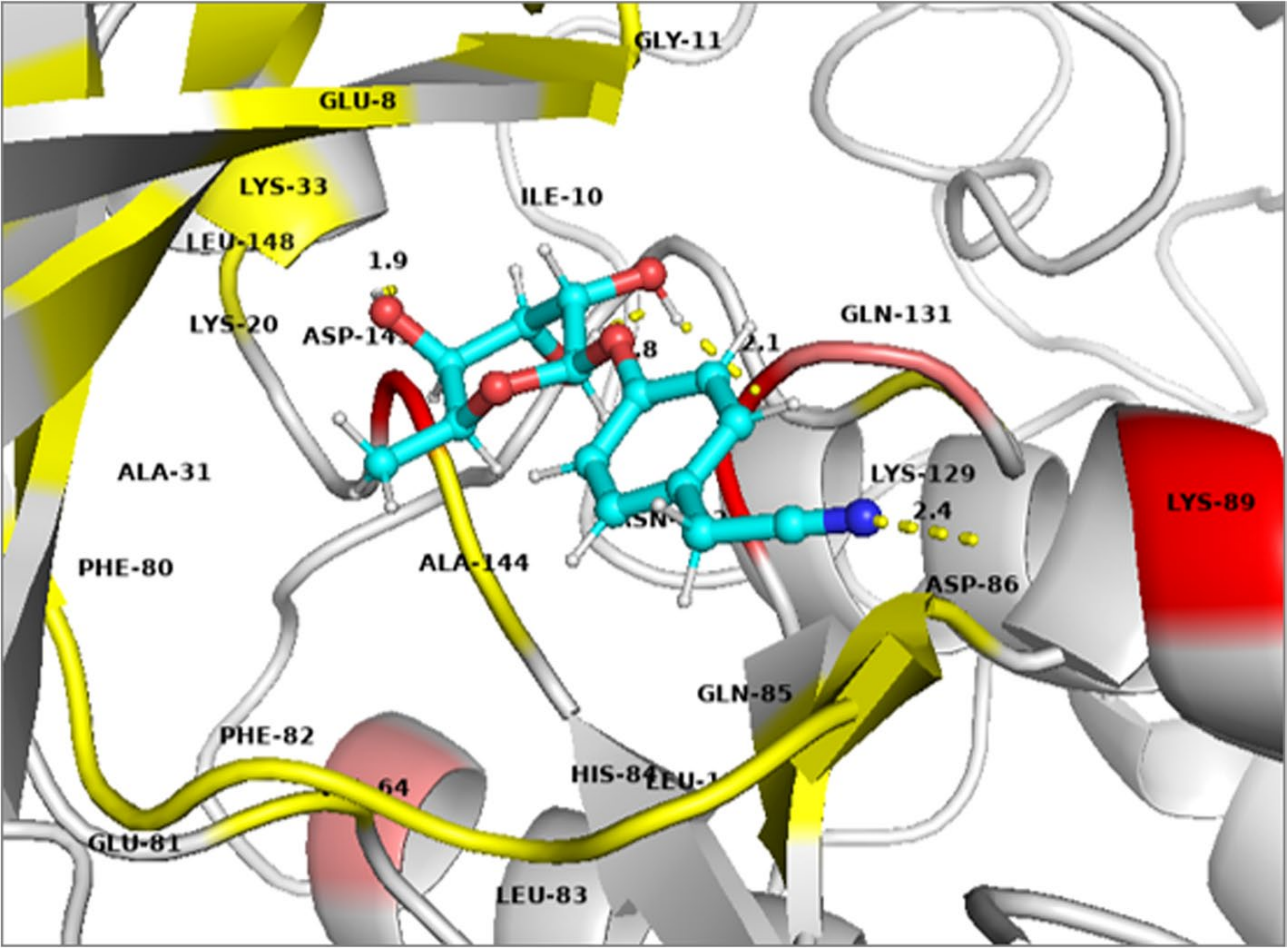
3D presentation of Niazirin [4] (cyan) in the active site of CDK-2 protein (PDB code: 2XMY)

Ellagic acid [3] showed polar interactions with Asp-145, Asp-86 and Leu-83 while non-polar interactions with Asn-132, Gln-131, Glu-12, His-84, Ile-10 and Lys-89 as shown in Fig. 6a. Kaempferol [5] showed polar interactions with Asp-145 and His-84 while non-polar contacts with Asn-132, Asp-86, Gln-131, Glu-81, Ile-10, Lys-89, Leu-83 and Leu-298 as shown in Fig. 6b. Ellagic acid [3] and Kaempferol [5] overlapping at the active site of CDK-2 protein is shown in Fig. 7. Niazirin (4) showed polar interactions with Asp-145, Asn-132, Gln-131 and Lys-89 while non-polar interactions with Asp-86, Glu-8, His-84, Ile-10, Lys-20, Lys-129 and Leu-148 as shown in Fig. 8

The residues with the least interactions include Leu-298, Glu-8, Lys-9, Lys-20 and Leu-148.

Asp-92, Glu-12, Gu-162, Thr-158, Thr-160, Thr-165, Tyr-159, Tyr-168, Trp-167, Lys-88, Lys-129, Val-163 and Val-164 were the residues involved in non-polar interactions in dinaciclib only as mentioned in Table 3.

**Table 3:** Polar and non-polar interacting residues with compounds in binding pocket of CDK-2 protein

Compound	Name	Interaction Type	Residues
**Reference**	Dinaciclib	Polar	Gln-131, Lys-9
		Non-polar	Asp-86, Asp-92, Glu-12, Glu-162, Gly-11, Thr-158, Thr-160, Thr-165, Tyr-159, Tyr-168, Trp-167, Lys-88, Lys-89, Lys-129, Val-163, Val-164
**1**	Chlorogenic acid	Polar	Asp-145, His-84, Lys-89, Ile-10
		Non-polar	Ala-31, Ala-144, Asn-132, Asp-86, Gln-131, Gln-85, Gly-11, Gly-13, Glu-8, Glu-81, Lys-9, Lys-33, Lys-20, Leu-148, Leu-298, Leu-134, Leu-83, Phe-80, Phe-82, Val-18, Val-64
**2**	Quercetin	Polar	Asp-145, His-84
		Non-polar	Ala-31, Ala-144, Asn-132, Asp-86, Gln-131, Gln-85, Gly-11, Gly-13, Glu-81, Ile-10, Lys-33, Lys-89, Leu-83, Leu-134, Leu-298, Phe-80, Phe-82, Val-18, Val-64
**3**	Ellagic acid	Polar	Asp-145, Asp-86, Leu-83
		Non-polar	Ala-31, Ala-144, Asn-132, Gln-131, Gln-85, Glu-81, Glu-12, Gly-11, Gly-13, His-84, Ile-10, Lys-33, Lys-89, Leu-134, Phe-80, Phe-82, Thr-14, Val-18, Val-64
**4**	Niazirin	Polar	Asp-145, Asn-132, Gln-131, Lys-89
		Non-polar	Ala-31, Ala-144, Asp-86, Gln-85, Glu-8, Gly-11, Gly-13, His-84, Ile-10, Lys-20, Lys-129 , Lys-33, Leu-83, Leu-134, Leu-148, Phe-80, Phe-82, Val-18, Val-64
**5**	Kaempferol	Polar	Asp-145, His-84
		Non-polar	Ala-31, Ala-144, Asn-132, Asp-86, Gln-131, Gln-85, Glu-81, Gly-11, Gly-13, Ile-10, Lys-33, Lys-89, Leu-134, Leu-298, Leu-83, Phe-80, Phe-82, Val-18, Val-64

The binding free energy of the ligand protein complex was evaluated by MM-GBSA method in Maestro 13.2 (Schrödinger, LLC, 2022.2) and the compounds with highest binding free energy in negative value were further analyzed in MD simulations using Maestro-Desmond v12.3 Schrödinger software for evaluating the stability of the complex.

#### Prime MM-GBSA

The prime MM-GBSA method was used to calculate the binding free energy of ligands and protein complex. All the docked poses were optimized using OPLS 2005 force field feature in prime and Generalized-Born/ Surface Area continuum solvent model was used to calculate energies of complex.

This analysis revealed the binding energy ∆G of dinaciclib) with CDK-2 protein as -36.21 kcal/mol in comparison with best docked ligand chlorogenic acid (1) -38.16 kcal/mol. Quercetin (2) had binding energy less than dinaciclib (reference) -34.02 kcal/mol. Ellagic acid (3) had the highest negative binding energy as -48.91 kcal/mol. Niazirin (4) had binding energy close to the top hit compound that is -38.65 kcal/mol. The binding energy of kaempferol (5) -31.92. Energy calculation by prime analysis gives relative energies of ligands. The ligands with the highest negative energies and binding affinity as mentioned in Table 4 were further selected for analysis in MD simulation.

**Table 4:** Prime MM-GBSA results of *Moringa Oleifera* compounds reported in literature

Ligand	Name	∆G Bind
Reference	Dinaciclib	-36.21
1	Chlorogenic acid	-38.16
2	Quercetin	-34.02
3	Ellagic acid	-48.91
4	Niazirin	-38.65
5	Kaempferol	-31.92

#### Molecular dynamic simulation

Molecular Dynamic (MD) simulations were carried out using Desmond Molecular Dynamics Simulation System to check the stability of ligands and optimization of complexes. The trajectories obtained from simulations were analyzed using RMSF and RMSD. RMSF was calculated to check flexibility of ligand and changes in its conformation upon binding. The RMSD measured the displacement of a selection of atoms over this time period. Molecular dynamic simulations were run at normal pressure and temperature for 100 ns and RMSD plots of complex were generated. The protein and ligand contacts were recorded as fraction plots for binding during the simulation.

**Fig. 9:**
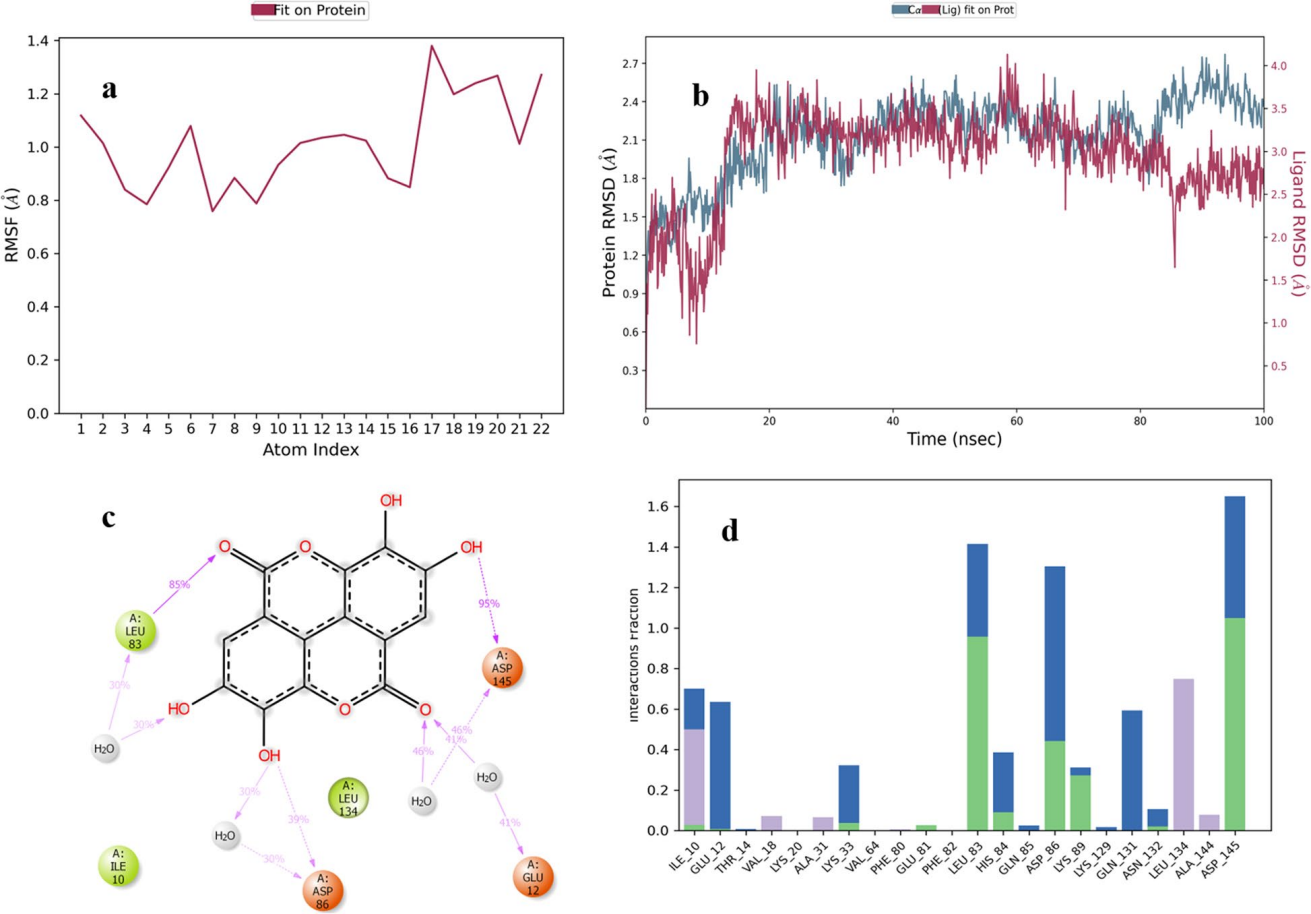
MD simulation studies of ellagic acid in complex with CDK-2 protein

The RMSF of ellagic acid is shown in Fig. 9a. The RMSD value of ellagic acid-protein complex was highest 4 Å (Fig. 9b). Asp-145 residue of protein forms H-bonds with ligand for 95% along with bridges of water for 46%, Leu-83 forms H-bonds with ligand for 85% along with bridges of water for 30% and Asp-86 forms H-bonds for 39% along with bridges of water for 30% of the simulation time. Similarly, Val-18, Ala-31, Ala-144, Ile-10 and Leu-134 show hydrophobic contacts for 10–50% of the time. Other amino acid residues for stabilizing the complex forming H-bonds and water bridges include His-84, Asn-132, Ile-10, Lys-89, Lys-33, Glu-81, Glu-12, Thr-14, Gln-85 and Lys-129 (Fig. 9d). Figure 9c shows a schematic diagram of several amino acid residues of CDK-2 involved in interactions with ellagic acid.

**Fig. 10:**
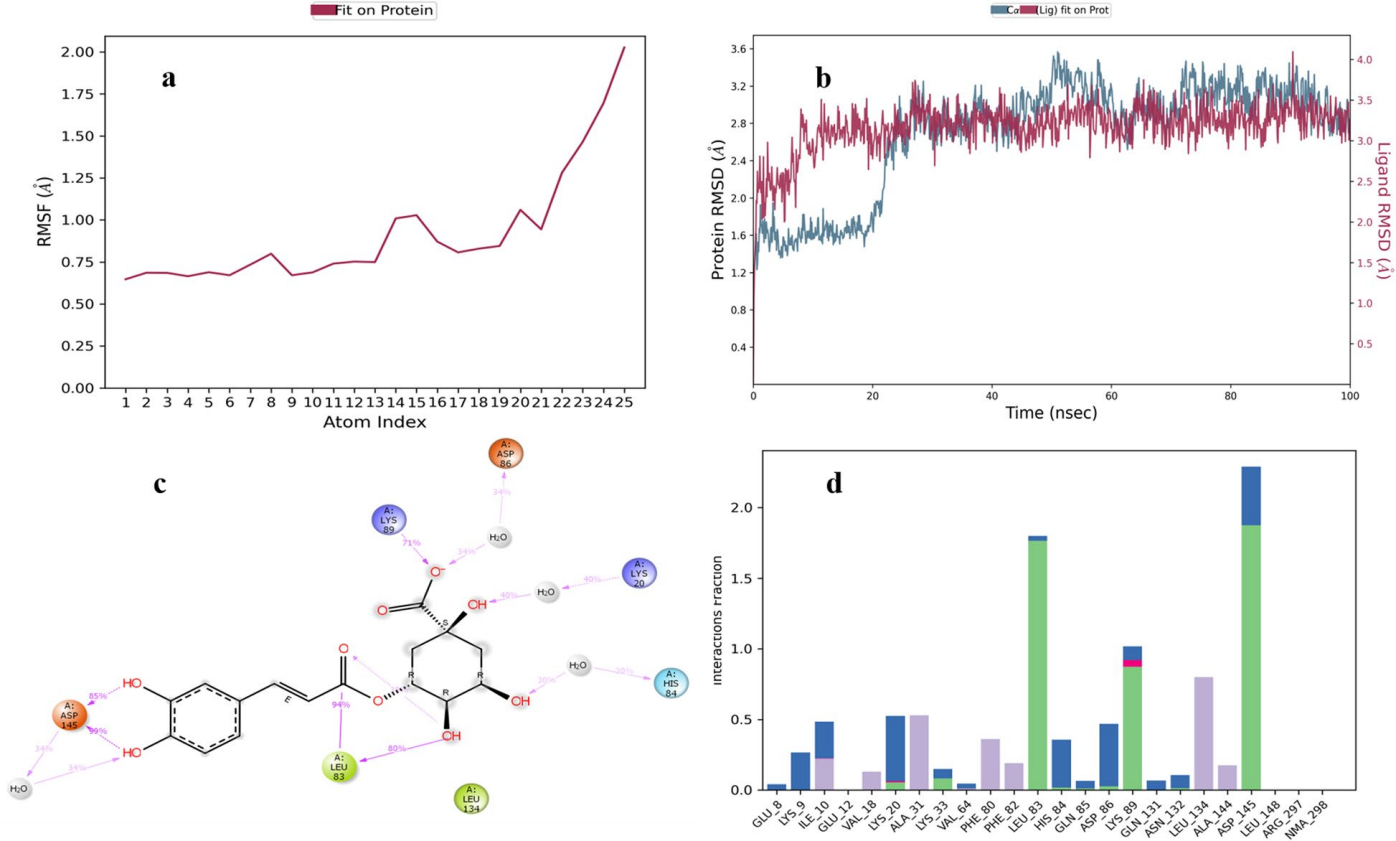
MD simulation studies of chlorogenic acid in complex with CDK-2 protein

The RMSF of chlorogenic acid is shown in Fig. 10a. In chlorogenic acid-protein complex equilibrium of system is attained within one sec and average RMSD value of 3.8 Å (Fig. 10b). Asp-145 residue forms H-bonds with ligand for 85–99% along with water bridges for 34%, Leu-83 and Lys-89 form H-bonds for 94% and 71%. Lys-20, Asp-86 and His-84 form H-bonds and bridges of water for 40%, 34% and 30% of the simulation time respectively. Similarly, Phe-80, Phe-82, Ala-31, Val-64, Leu-134, Ile-10, Ala-144 and Val-18 show hydrophobic contacts for 10–20% of the simulation time. The residues involved in ionic interaction include Lys-89, Ile-10 and Lys-20. Other amino acid residues for stabilizing the complex forming water bridges include Asn-132, Glu-8, Lys-9, Lys-20, Lys-33, Gln-85, and Gln-131 (Fig. 10d). Figure 10c shows a schematic diagram of several amino acid residues of CDK-2 involved in interaction with chlorogenic acid.

**Fig. 11:**
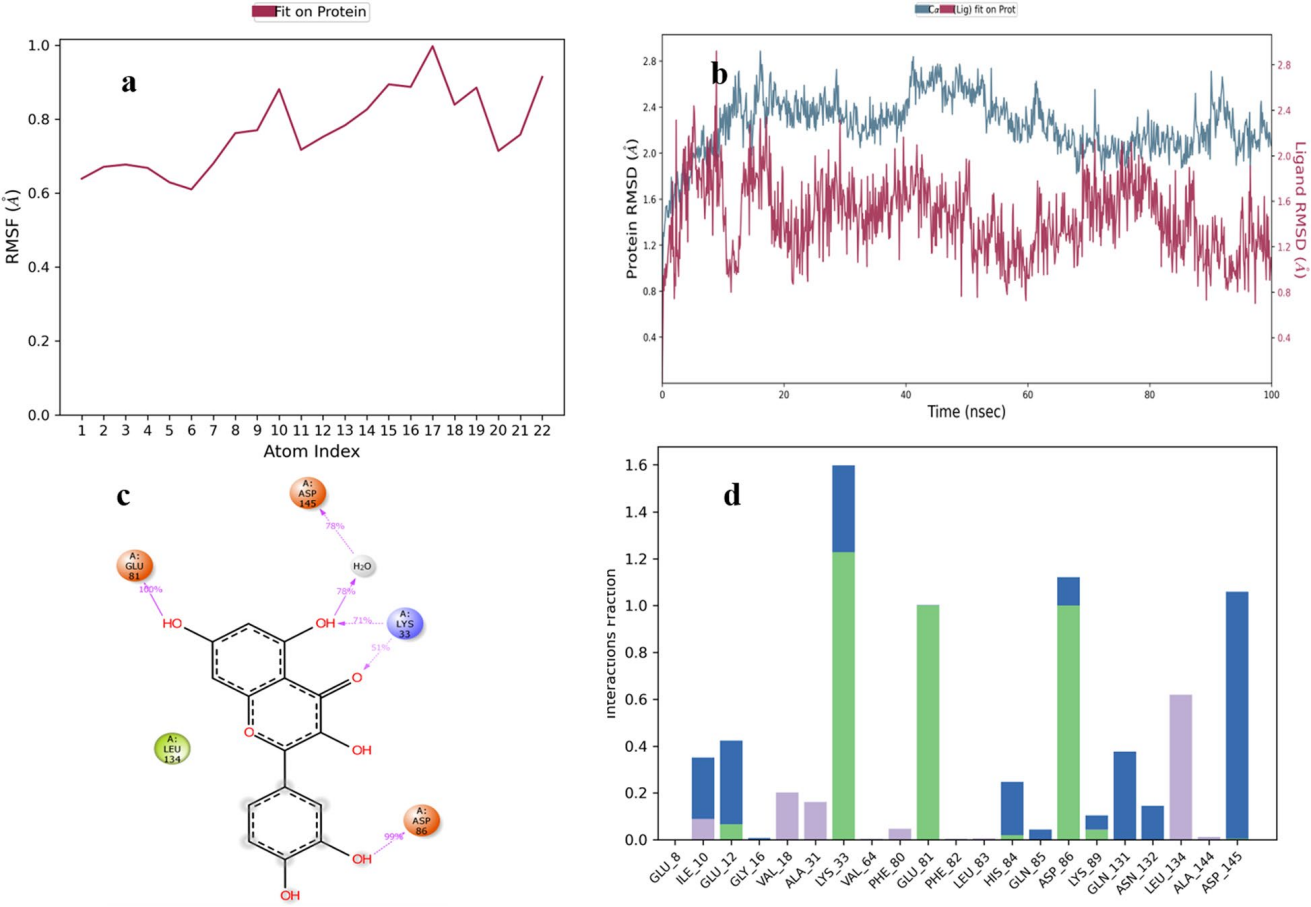
MD simulation studies of quercetin in complex with CDK-2 protein

The RMSF of quercetin is shown in Fig. 11a. In quercetin-protein complex equilibrium of system is attained within one sec and average RMSD value of 2.7 Å (Fig. 11b). Glu-81 and Asp-86 residues of protein form H-bonds with ligand for 100% and 99% of the time respectively. Asp-145 forms H-bonds for 78% of the time along with water bridges. Lys-33 forms hydrogen bonds for 51–71% of the simulation time. Similarly, Phe-80, Ala-31, Ile-10, Val-18, Phe-82, Leu-83, Ala-144 and Leu-134 show hydrophobic contacts for 10–30% of the total simulation time. Other amino acid residues for stabilizing the complex forming water bridges and hydrogen bonds include Asp-145, Asn-132, Gly-16, Glu-12, His-84, Ile-10, Lys-89, Gln-85, and Gln-131 (Fig. 11d). Figure 11c shows a schematic diagram of several amino acid residues of CDK-2 involved in interaction with quercetin.

#### ADME (absorption, distribution, metabolism and excretion) Prediction

Top ranked compounds from molecular docking were analyzed for their pharmacokinetic properties using Swiss ADME server. ADME server predicts the physicochemical properties of compounds including molecular weight, partition co-efficient of octanol/water (log P_**o/w**_) & relative absorption in intestine included in Table 5.

**Table 5:** Index of ADME properties to predict drug-likeliness of the ligands

Ligands	Mol. formula	Mol. Wt g/mol	Rotatable Bonds	H-bond Acceptors	H-bond Donors	Log P_o/w_	GI Absorption
Chlorogenic acid	C_16_H_18_O_9_	354.31	5	9	6	-0.39	Low
Quercetin	C_15_H_10_O_7_	302.23	1	7	5	1.23	High
Ellagic acid	C_14_H_6_O_8_	302.19	0	8	4	1.00	High
Niazirin	C_14_H_17_NO_5_	279.29	3	6	3	0.32	High
Kaempferol	C_15_H_10_O_6_	286.24	1	6	4	1.58	High

According to five rules of Lipinski (RO5) of likeliness of drug for consideration in pre-clinical studies a good drug candidate should have a molecular weight less than or equal to 500 Dalton, rotatable bonds should be less than or equal to 10, hydrogen bond acceptors should be less than or equal to 10 in number, also hydrogen bond donors should be less than or equal to 5 in number and log value (P _**o/w**_) should be ≤ 5 [40–42].

All of the compounds analyzed are good candidates for drug design, follow Lipinski rule and show no violations except chlorogenic acid as shown in Table 5. Chlorogenic acid exhibits one violation. Number of H-bond donors is 6 in chlorogenic acid. Also, it exhibits low GI absorption compared to other compounds. However, it can be enhanced synthetically by modifying physical properties that will improve the permeability, lipophilicity and absorption of the compounds.

#### Toxicity Prediction

The toxicological properties of compounds were predicted by webserver Pro-Tox II with results summarized in Table 6. The oral toxicity of compounds predicted ranged from 159 mg/kg to 5000 mg/kg. Quercetin (159 mg/kg) was the only compound that belonged to toxicity class III, toxic if swallowed. The compound ellagic acid (2991 mg/kg) belonged to toxicity class IV, harmful if swallowed. All the other compounds such as chlorogenic acid (5000 mg/kg), niazirin (3750 mg/kg), and kaempferol (3919 mg/kg) belonged to class v, could be harmful if swallowed. However, none of the screened compounds were predicted in severe toxic class that is fatal (Class I or II). So, they can be used as lead compounds in the treatment of breast cancer.

**Table 6:** Prediction of acute oral toxicity and toxicity class of ligands

Ligands	LD50 predicted in Rodent (mg/kg)	Toxicity Class
Chlorogenic acid	5000	V
Quercetin	159	III
Ellagic acid	2991	IV
Niazirin	3750	V
Kaempferol	3919	V

Class I: fatal if swallowed (LD50 ≤ 5), Class II: fatal if swallowed (5 < LD50 ≤ 50). Class III: toxic if swallowed (50 < LD50 ≤ 300). Class IV: harmful if swallowed (300 < LD50 ≤ 2000). Class V: may be harmful if swallowed (2000 < LD50 ≤ 5000). Class VI: non-toxic (LD50 > 5000)

### *In-vitro* results

#### Effect of Fraction A (petroleum ether) on Mcf-7 cell lines

**Fig. 12:**
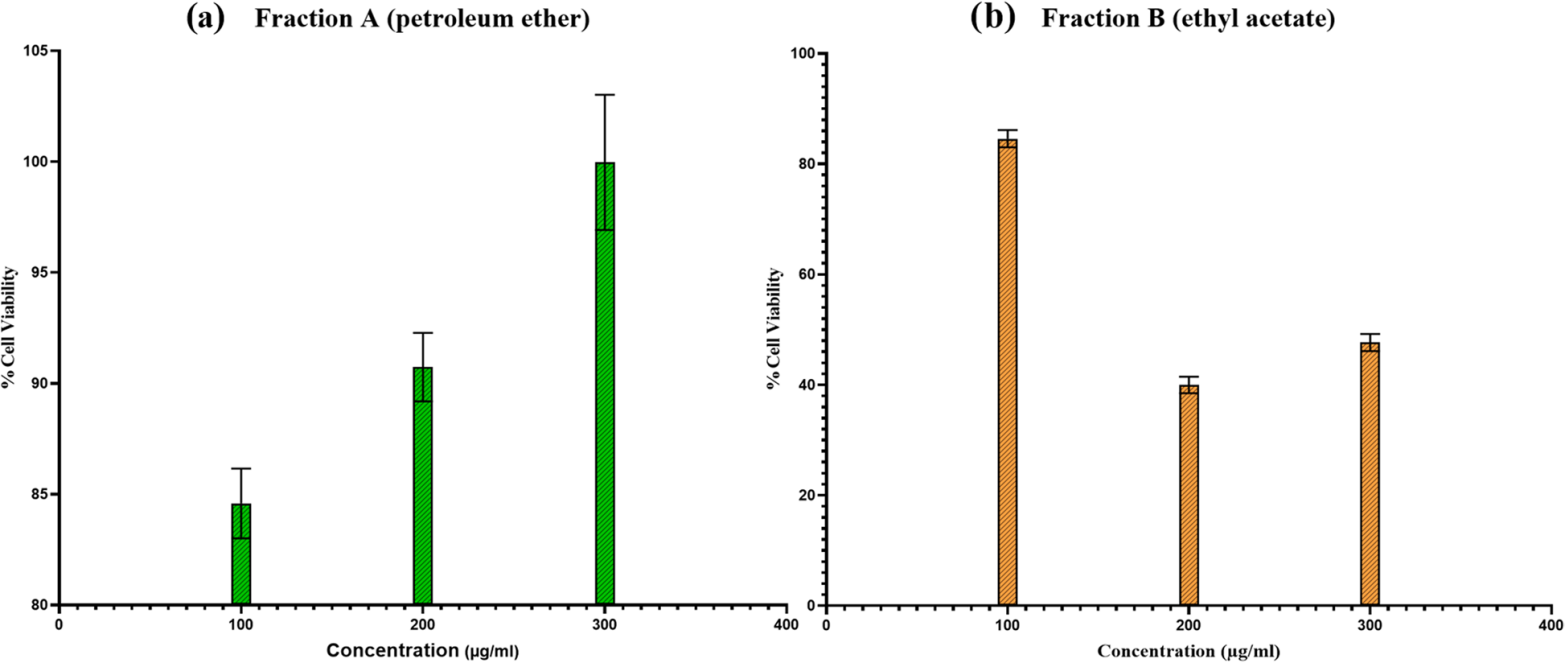
Viability of Mcf-7 cells following 24 h fraction A (**a**) and fraction B (**b**) exposures. At concentrations: 100 µg/mL, 200 µg/mL,300 µg/mL

The results of MTT assay using petroleum ether fraction of *Moringa oleifera* leaves extract are shown in Fig. 12a. After 24 h, results were compared with control and no significant reduction was observed in cell viability, rather an exposure of 300 µg/mL of extract resulted in an increase in cell viability by 99.9% and other two dilutions (100 µg/mL, 200 µg/mL) also showed enhanced proliferation as 84.6% and 90.7% respectively.

#### Effect of Fraction B (ethyl acetate) on Mcf-7 cell lines

The results of MTT assay using ethyl acetate fraction of *Moringa oleifera* leaves extract are shown in Fig. 12b. After 24 h, results were compared with control significant reduction was observed in cell viability at higher concentrations of extract, 40% at 200 µg/mL and 47.7% at 300 µg/mL respectively. However, no significant reduction was observed at 100 µg/mL and cell viability was increased by 84.6%.

Though it was observed that both extracts exhibited slight cytotoxicity after 24 h, while Mcf-7 cells showed enhanced proliferation. The findings of MTT assay indicate that extracts of *Moringa oleifera* have not shown any significant anticancer effect on Mcf-7 cell lines.

#### Statistical results

The statistical significance was calculated using one way analysis of variance (ANOVA) and value of *P* < 0.05 was taken as statistically significant. The *p* value for petroleum ether data was found to be 0.0024 and for ethyl acetate it was 0.05. So, the statistical results for petroleum ether fraction were found to be more significant.

## Discussion

Due to emerging drug resistance in breast cancer treatment, there is a need for drug design & development involving target specific therapies. Although various drugs including palbociclib, ribociclib & abemaciclib have been recognized by FDA United States but they are potent inhibitors of only cyclin dependent kinase 4 & 6 enzymes in cell cycle [43, 44]. Researchers are working on developing target specific CDK2 inhibitor in treating ER positive breast cancer. Dinaciclib is in clinical trials & a novel research drug targeting CDK2 specifically. It has shown safety profiles in pre-clinical studies against various hematological malignancies targeting CDK2, CDK1, CDK5 and CDK9. Still, it in is clinical investigations for treating cancer [24, 45]. Computational approach in drug design have gained importance in drug development process in the recent years. It is less time consuming and have low cost as compared to traditional process of drug development [46, 47]. By using this approach of computational or *in-silico* drug design we determined the anticancer potential of *Moringa oleifera* in ER + breast cancer targeting CDK-2 enzyme & evaluated our results using Maestro v13.2, Schrödinger. The compounds of *Moringa oleifera* reported in the literature were docked using Glide Docking program. The interactions of the top hit compounds chlorogenic acid (1), quercetin (2), ellagic acid (3) were visualized using PYMOL-2.5.2. Molecular dynamic simulations were carried to evaluate stability of best docked compounds. The anticancer potential of our plant extract was validated *in-vitro* using Mcf-7 cell lines and MTT assay technique in experimental work. *Moringa oleifera* is a rich source of several compounds, such as flavonoids, phenolic acids, alkaloids, phytosterols, minerals, vitamins & many organic acids. It has many therapeutic, nutritional and industrial applications [48]. The compounds of *Moringa oleifera* reported in literature including 1,3 dibenzyl urea, moringine, moringinine, niazirin, niazirinin, niaziminin, niazimicin, benzyl isothiocyanate, phenolic acids, kaempferol, quercetin, isoquercetin, rutin, lutein, beta carotene, vitamin B2, nicotinic acid, pyridoxine, folic acid, tocopherol, ascorbic acid, methionine, cystine, tryptophan, lysine, β-sitosterol, stigmasterol, octacosanoic acid,4-hydroxymellein, aurantiamide acetate, & vanillin [49] were screened for their anticancer activity targeting CDK2 protein employing *in-silico* techniques & interactions were visualized using PYMOL 2.5.2.

Dinaciclib was used as reference drug. Out of 36 compounds chlorogenic acid (1), quercetin (2), ellagic acid (3), niazirin (4) & kaempferol (5), had better interactions with the protein & could be the promising inhibitors of CDK2 protein. The interaction and docking scores of other compounds are provided in supplementary information. The binding affinity of the dinaciclib (reference) was-5.445 kcal/mol. Molecular docking revealed that all the ligands showed good binding with target while chlorogenic acid (1), quercetin (2), ellagic acid (3) had binding affinity of -9.119, -7.501 and -7.138 kcal/mol highest negative than the dinaciclib (reference ligand). Dinaciclib had only two conventional hydrogen bonds. Chlorogenic acid (1) interacted with target protein forming five conventional & one aromatic H-bond. It also exhibited salt bridges & residue involved was Lys-89. Chlorogenic acid (an ester of caffeic and quinic acid) is important phenolic acid. In previous studies conducted on animal models chlorogenic acid has shown to exhibit hepatoprotective, antihyperlipidemic & anti-tumor activities [50–52]. It is an important dietary polyphenol abundant in coffee beans & exhibits anticarcinogenic, hepatoprotective, neuroprotective & cardioprotective effects. It plays a vital role in regulating the glucose & lipids metabolism thus treats related disorders such as obesity, diabetes & cardiovascular diseases [53–55]. Quercetin (2) binds with target forming two conventional & one aromatic H-bond. It is a flavonol present in many fruits & vegetable in the glycoside form [56]. Its anticancer potential is supported by previous studies conducted on phenolic compounds of *Moringa oleifera* showing good affinity with BAX (pro-apoptotic) proteins [55]. This study shows that quercetin has better binding affinity with CDK-2 protein as compared to BRCA-1 gene [57]. Ellagic acid (3) interacted with protein forming for conventional & one aromatic H-bond. It is a natural phenolic acid found in fruits which exhibited antiproliferative & apoptotic activities in various cancer cell lines through several mechanisms [58]. In an *in-silico* study conducted on dietary phytoconstituents utilizing CDK6 as target proteins, ellagic acid was reported as potent CDK6 inhibitor inducing apoptosis of cancer cells. It downregulates the expression of CDK6 & could be further evaluated for anticancer therapies [59].The binding affinities of niazirin (4) & kaempferol (5) were -6.835 & -6.717 kcal per mol respectively. In niazirin four conventional H-bonds as well as pi-pi stacking was observed between Phe-4 and Tyr-77 residues in maestro. Kaempferol (5) formed two conventional & three aromatic H-bonds. Niazirin is a nitrile glycoside obtained from leaves of *Moringa oleifera* in abundance & reported to have many antitumor & antimicrobial activities [60, 61]. In previous literature kaempferol, aglycone flavonoid, showed bad affinity for BRCA-1 genes with score -3.661 kcal/mol but reported to have anticancer potential in breast & many other cancers [62]. Various studies on *Moringa oleifera* concluded its antiproliferative effects on Mcf-7 cells [63–66].

The method of MM-GBSA was used to check relative free binding energies of ligands choosing best docked poses from the molecular docking. The ∆G bind for dinaciclib (reference ligand) was -36.21 kcal/mol. Ellagic acid (3), chlorogenic acid (3) & niazirin (4) had highest negative free binding energies as -48.91 kcal/mol, -38.16 kcal/mol & -38.65 kcal/mol respectively as compared to dinaciclib. MD simulation was run 100 ns for the top hit compounds chlorogenic acid, quercetin & ellagic acid to evaluate the stability. All of them were stable & average RMSD values were 3.8 Å, 4 Å & 2.7 Å respectively. Ellagic acid had the highest RMSD value. All three compounds showed hydrogen bonds as well as hydrophobic interactions with active sites of CDK-2 protein. Chlorogenic acid exhibited ionic interactions as well. The ADME & toxicity profiles were determined through Swiss & ProTox II servers. All the best hit compounds followed the Lipinski rules of drug & showed no violation but chlorogenic acid exhibited low GI absorption that can be improved by modifying physical properties of the compound. chlorogenic acid [1], niazirin (4) & kaempferol (5) belonged to toxicity class v: could be harmful if swallowed (2000 < LD50 ≤ 5000). Ellagic acid (3) belonged to toxicity class iv: harmful if swallowed (300 < LD50 ≤ 2000). Quercetin (2) belonged to toxicity class III: toxic if swallowed (50 < LD50 ≤ 300). None of the compounds belonged to severe toxic class & are safe candidates for treatment of breast cancer. The MTT assay was performed using Mcf-7 cell lines to validate the anticancer potential of compounds present in our extract. Petroleum ether & ethyl acetate fractions of *Moringa oleifera* were prepared using ethanolic extract. Three dilutions (100 µg/mL, 200 µg/mL, 300 µg/mL) of both fractions were incubated for 24 h in triplicate using 96 well plates. The absorbance was determined using Elisa reader & percentage of cell viability was calculated. It was observed that ethyl acetate fraction 2 showed better results & antiproliferative activity with increasing concentration. The percentage of cell viability was 84.6% at 100 µg/mL, 40% at 200 µg/mL and 47.7% at 300 µg/mL. The significant antiproliferation activity was observed at 200 µg/mL for ethyl acetate fraction. So, the optimum dose of ethyl acetate fraction with antiproliferative activity was 200 µg/mL. Petroleum ether fraction showed no significant antiproliferative activity after 24 h rather Mcf-7 cells showed enhanced proliferation at higher concentration 300 µg/mL and cell viability was 99.9%. However, slight cytotoxic effect was observed at 100 µg/mL with percentage cell viability of 84.6%. The findings of our *in-vitro* analysis suggest further investigation to determine the optimum doses of our extracts with enhanced antiproliferative activity. These could be the lead compounds as they are non-toxic & will have better bioavailability if used in drug design. Our study has certain limitations concerning the use of normal cell lines for comparison with the cancer cell lines. Also, we could have done *in-vivo* analysis using the dinaciclib dosage available in market for validation of our computational work. In future the *in-vitro* analysis can be done using CDK-2 enzymes as no enzymes were available for this study due to lack of resources.

## Conclusion

In conclusion, combination of molecular docking and molecular mechanics as well as molecular dynamic simulations, we recognized inhibitors potent for CDK-2 protein in treating estrogen receptor positive breast cancer.

In this work, by analyzing various interactions between ligands and CDK-2 protein key amino acid residues were identified. MMGBSA method was employed to check relative energies of ligands choosing best poses from docking and dynamic simulations were performed to validate the results. Analysis of dynamic simulations verified that all of the best compounds were stable over simulation time of 100 ns. The top hit compounds included chlorogenic acid, quercetin and ellagic acid. Ellagic acid being the most stable in simulation results. Overall, all these compounds could be the potential candidates as inhibitors of CDK-2 protein. The cytotoxic effects of our potential inhibitors were verified by *in-vitro* studies using cancer cell lines (Mcf-7) in MTT assay technique. The *in-vitro* studies conducted for the different fractions of *Moringa oleifera* revealed no significant anticancer activity because of the absence of potent anticancer compounds from our plant extract. In future we can evaluate the potential of our best hit compounds against other targets including CDK-4/6 and other enzymatic pathways of cell cycle to analyze the binding interactions and stability using *in-silico* techniques. Furthermore, the anticancer potential could be verified using *in-vivo* models as well.

### Supplementary Information

**Additional file 1.**

## Data Availability

The data generated during and/or analysed during the current study are available from the corresponding author on reasonable request.
