# Supplementary material for: The anticancer potential of chemical constituents of Moringa oleifera targeting CDK-2 inhibition in estrogen receptor positive breast cancer using in-silico and in vitro approches
Source: BMC Complement Med Ther. 2023 Nov 4;23:396. doi: 10.1186/s12906-023-04198-z (PMC10625284; doi:10.1186/s12906-023-04198-z)
Supplement: Supplementary file 1 — Additional file 1. [file 12906_2023_4198_MOESM1_ESM.zip › Supplementary Informtaion-Rida.docx]

# Supplementary Information

**Exploring the anticancer potential of chemical constituents of *Moringa oleifera* targeting CDK-2 inhibition in estrogen receptor positive breast cancer using *in-silico* and *in vitro* approches**

Rida Sultan^a^, Abrar Ahmed^a^†, Li Wei^b,^ Hamid Saeed^a^, Muhammad Islam^a^, Muhammad Ishaq^c^

1. Punjab University College of Pharmacy, Faculty of Pharmacy, University of the Punjab, Lahore, 54590, Pakistan
2. Zhongshan Institute for Drug Discovery, Shanghai Institute of Materia Medica, Chinese Academy of Sciences (CAS), Zhongshan 528400, P.R. China
3. Institute of Social and Cultural Studies, University of the Punjab, Lahore, 54590, Pakistan

*Correspondence: Dr. Abrar Ahmed

E-mail address: [abrar.pharmacy@pu.edu.pk](mailto:abrar.pharmacy@pu.edu.pk)

Tel.: +92 3335748738

## Supplementary Table

[Table S1: Interaction analysis of compounds of Moringa oleifera](#_Toc117452194)

## Supplementary Figures

[Figure S2: Two dimensional structures of compounds of Moringa oleifera](#_Toc117453167)

[Figure S3: Two dimensional structures of compounds of Moringa oleifera](#_Toc117453168)

[Figure S4: Complex of rutin (1) with CDK-2 protein showing polar contacts](#_Toc117453169)

[Figure S5: Complex of rutin (1) with CDK-2 protein showing non- polar contacts](#_Toc117453170)

[Figure S6: Complex of moringinine (2) with CDK-2 protein showing polar contacts](#_Toc117453171)

[Figure S7: Complex of moringinine (2) with CDK-2 protein showing non-polar contacts](#_Toc117453172)

[Figure S8: Complex or moringine (3) with CDK-2 protein showing polar contacts](#_Toc117453173)

[Figure S9: Complex of moringine (3) with CDK-2 protein showing non-polar contacts](#_Toc117453174)

[Figure S10: Complex of 4(4’-o-acetyl-alpha-l-rhamnosyloxy) Benzyl isothiocyanate (4) with CDK-2 protein showing polar contacts](#_Toc117453175)

[Figure S11: Complex of 4(4’-o-acetyl-alpha-l-rhamnosyloxy) Benzyl isothiocyanate (4) with CDK-2 protein showing non-polar contacts](#_Toc117453176)

[Figure S12: Complex of vitamin B2 (5) with CDK-2 protein showing polar contacts](#_Toc117453177)

[Figure S13: Complex of vitamin B2 (5) with CDK-2 protein showing non-polar contacts](#_Toc117453178)

[Figure S14: Complex of niazimicin (6) with CDK-2 protein showing polar contacts](#_Toc117453179)

[Figure S15: Complex of niazimicin (6) with CDK-2 protein showing non-polar contacts](#_Toc117453180)

[Figure S16: Complex of ferulic acid (7) with CDK-2 protein showing polar contacts](#_Toc117453181)

[Figure S17: Complex of ferulic acid (7) with CDK-2 protein showing non-polar contacts](#_Toc117453182)

[Figure S18: Complex of isoquercetin (8) with CDK-2 protein showing polar contacts](#_Toc117453183)

[Figure S19: Complex of isoquercetin (8) with CDK-2 protein showing non-polar contacts](#_Toc117453184)

[Figure S20: Complex of benzyl-isothiocyanate (9) with CDK-2 protein showing polar contacts](#_Toc117453185)

[Figure S21: Complex of benzyl-isothiocyanate (9) with CDK-2 protein showing non-polar contacts](#_Toc117453186)

[Figure S22: Complex of O-ethyl-4-(alpha-l-rhamnosyloxy) benzyl carbamate (10) with CDK-2 protein showing polar contacts](#_Toc117453187)

[Figure S23: Complex of O-ethyl-4-(alpha-l-rhamnosyloxy) benzyl carbamate (10) with CDK-2 protein showing non-polar contacts](#_Toc117453188)

[Figure S24: Complex of folic acid (11) with CDK-2 protein showing polar contacts](#_Toc117453189)

[Figure S25: Complex of folic acid (11) with CDK-2 protein showing non-polar contacts](#_Toc117453190)

[Figure S26: Complex of dl-alpha tocopherol (12) with CDK-2 protein showing polar contacts](#_Toc117453191)

[Figure S27: Complex of dl-alpha tocopherol (12) with CDK-2 protein showing non-polar contacts](#_Toc117453192)

[Figure S28: Complex of stigmasterol (13) with CDK-2 protein showing polar contacts](#_Toc117453193)

[Figure S29: Complex of stigmasterol (13) with CDK-2 protein showing non-polar contacts](#_Toc117453194)

Table S1: Interaction analysis of compounds of Moringa oleifera

| **Ligand** | **Name** | **XP-score** | **XP H-bond** | **Glide energy** | **Glide Ligand efficiency** |
| --- | --- | --- | --- | --- | --- |
|  | Rutin | -5.534 | -2.831 | -44.372 | -0.128 |
|  | Moringinine | -5.485 | -0.686 | -18.384 | -0.682 |
|  | Moringine | -5.427 | -2.079 | -46.288 | -0.270 |
|  | 4(4’-o-acetyl-alpha-l-rhamnosyloxy) Benzyl isothiocyanate | -5.141 | -2.151 | -51.193 | -0.210 |
|  | Vitamin B2 | -4.874 | -1.586 | -46.162 | -0.180 |
|  | Niazimicin | -4.550 | -2.369 | -41.811 | -0.190 |
|  | Ferulic acid | -4.455 | -0.950 | -27.900 | -0.318 |
|  | Isoquercetin | -4.267 | -1.949 | -32.878 | -0.128 |
|  | Benzyl-isothiocyanate | -4.256 | -0.546 | -26.220 | -0.414 |
|  | O-ethyl-4-(alpha-l-rhamnosyloxy) benzyl carbamate | -3.584 | -2.235 | -34.057 | -0.149 |
|  | Folic acid | -3.483 | -1.999 | -45.660 | -0.109 |
|  | dl-alpha tocopherol | -3.471 | -0.350 | -37.285 | -0.112 |
|  | Stigmasterol | -3.344 | -0.254 | -31.791 | -0.111 |

Figure S2: Two dimensional structures of compounds of Moringa oleifera

Figure S3: Two dimensional structures of compounds of Moringa oleifera

**
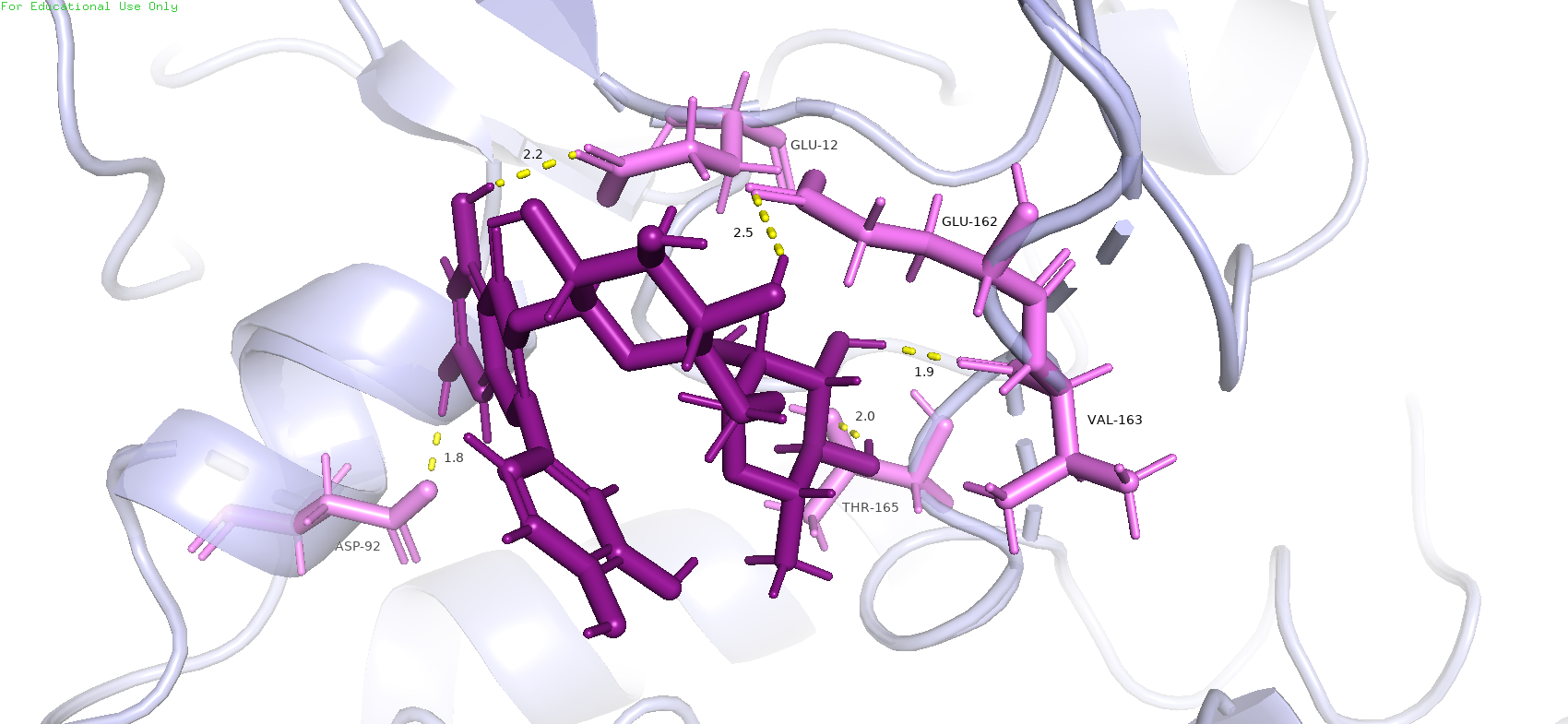
**

Figure S4: Complex of rutin (1) with CDK-2 protein showing polar contacts

**
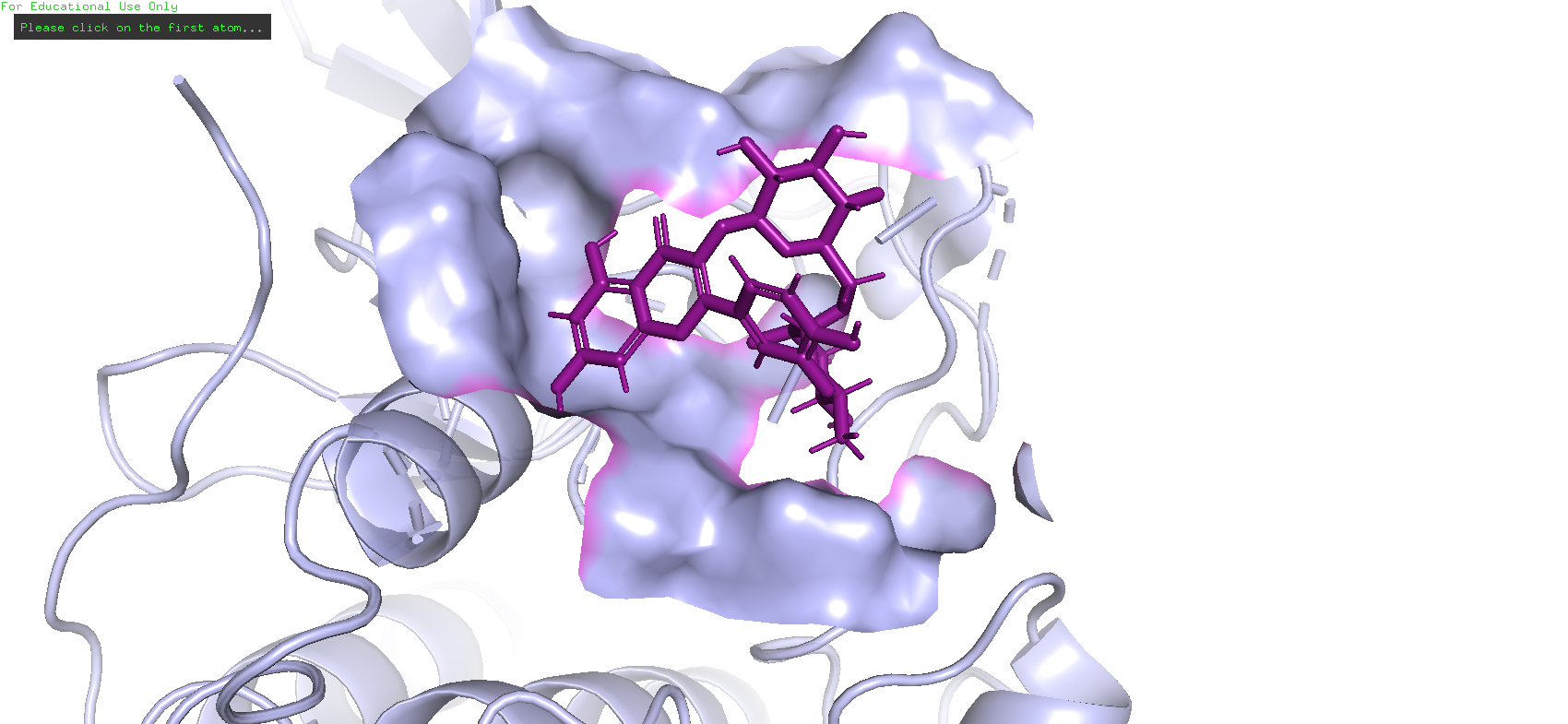
**

Figure S5: Complex of rutin (1) with CDK-2 protein showing non- polar contacts


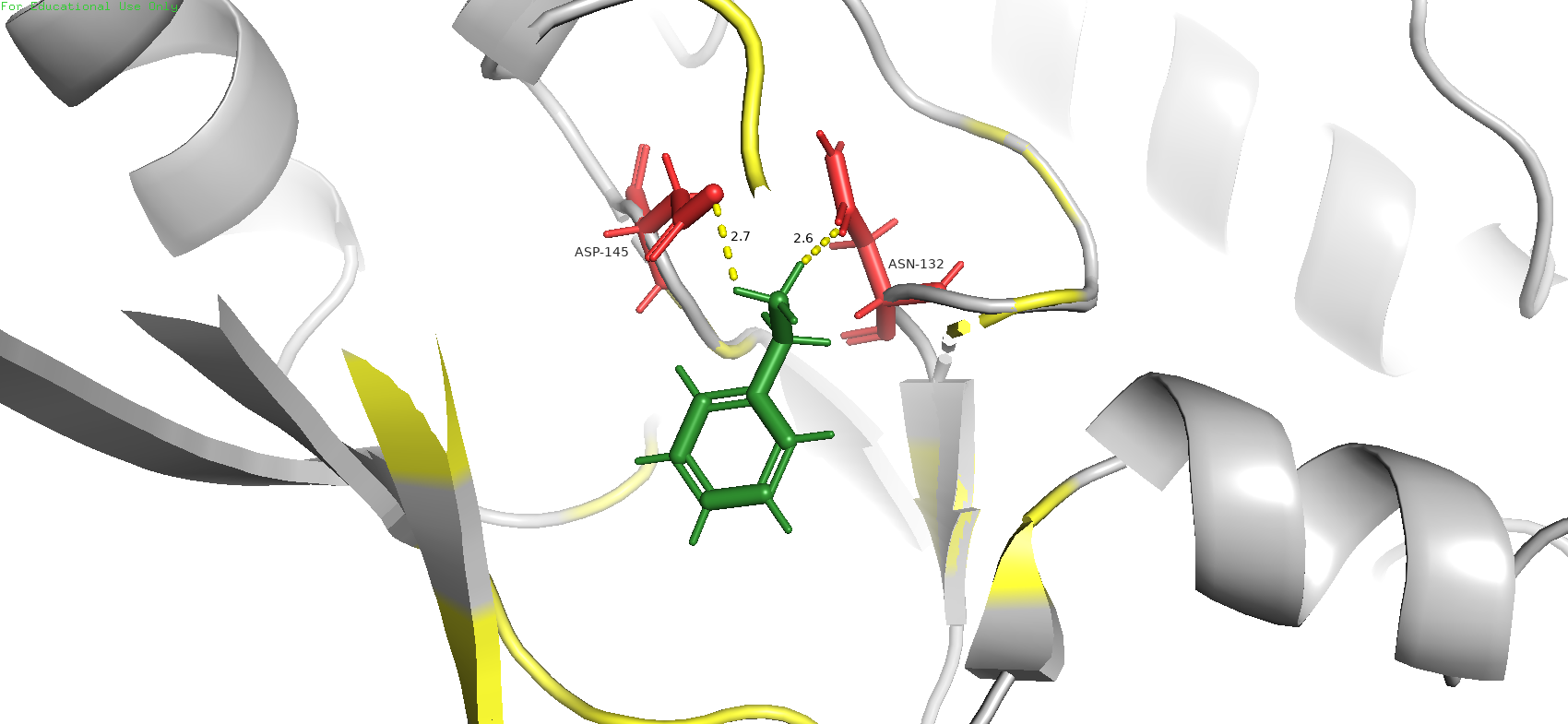


Figure S6: Complex of moringinine (2) with CDK-2 protein showing polar contacts


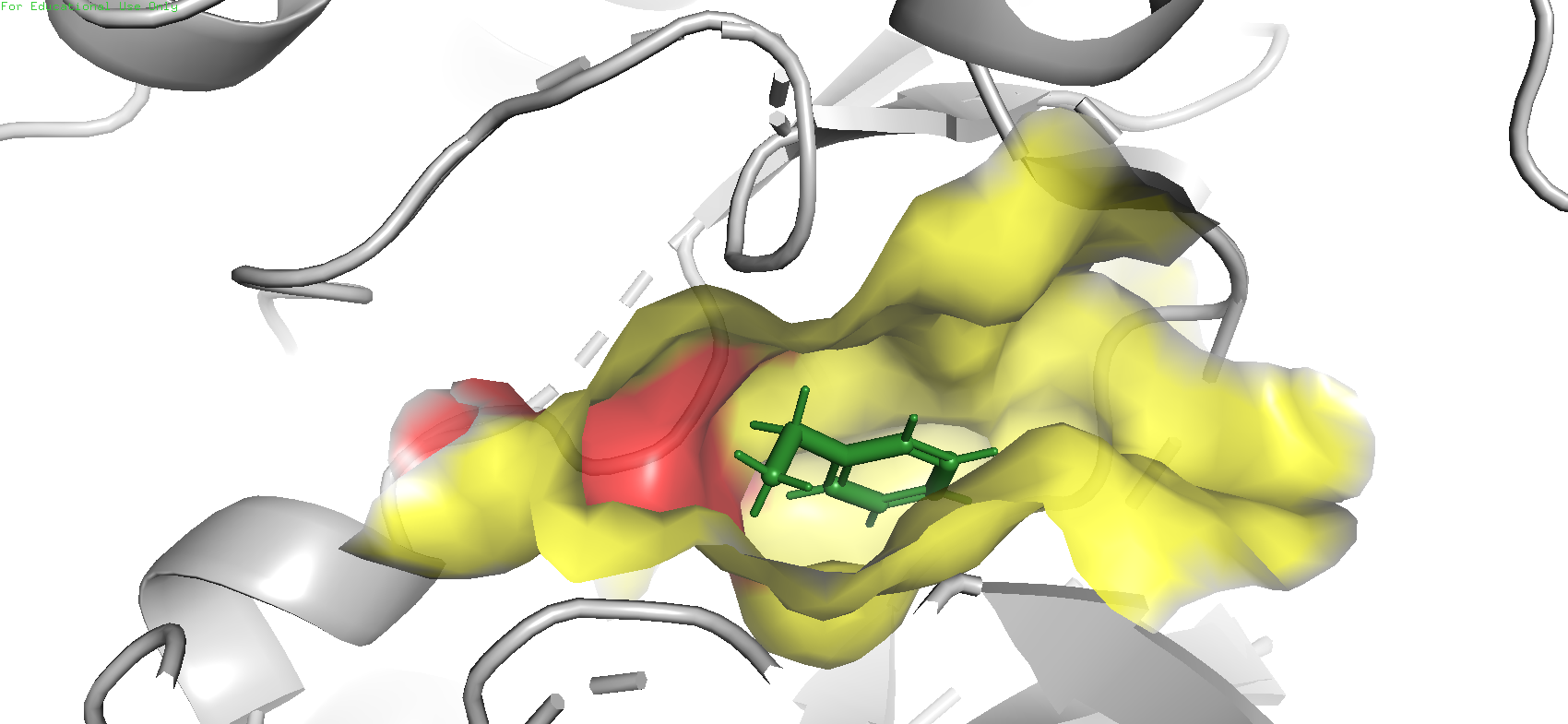


Figure S7: Complex of moringinine (2) with CDK-2 protein showing non-polar contacts


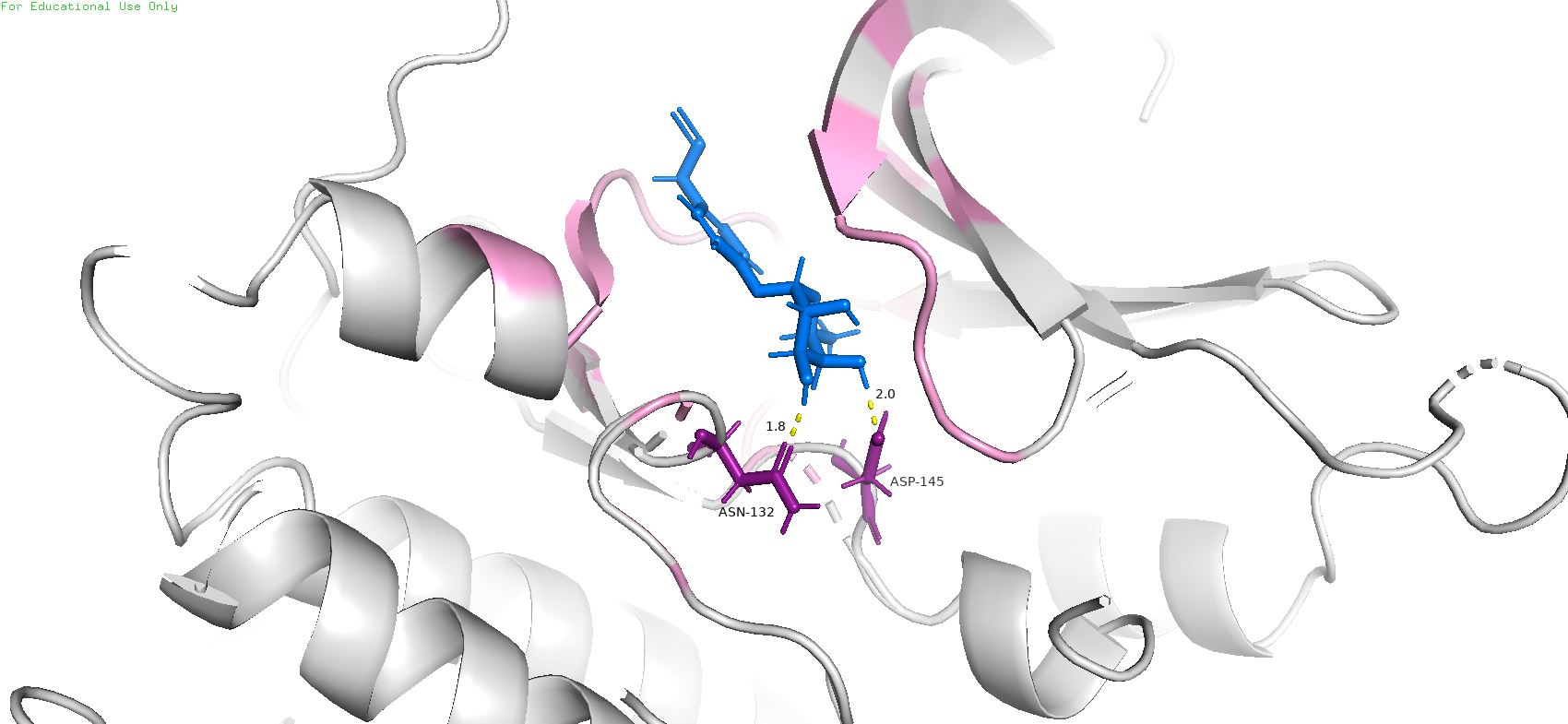


Figure S8: Complex or moringine (3) with CDK-2 protein showing polar contacts


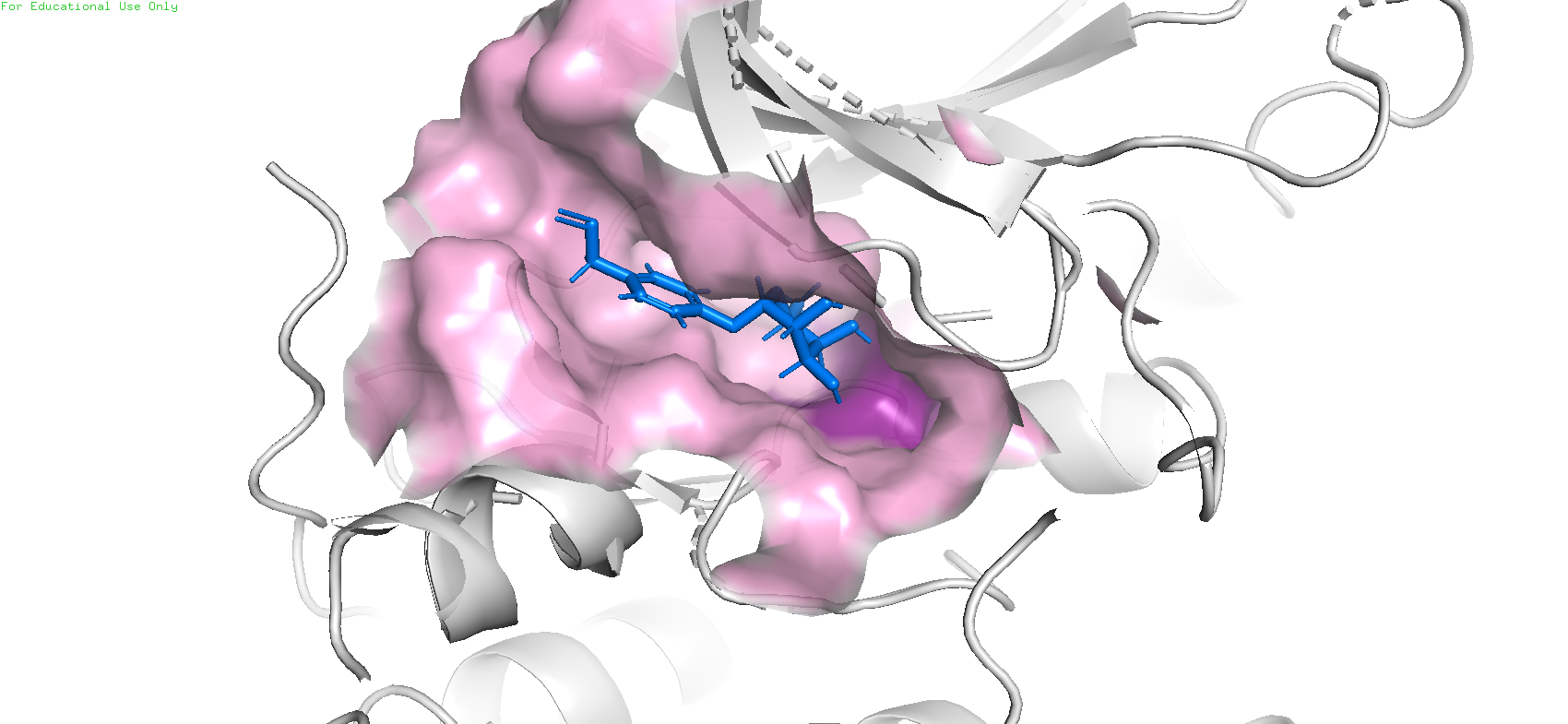


Figure S9: Complex of moringine (3) with CDK-2 protein showing non-polar contacts


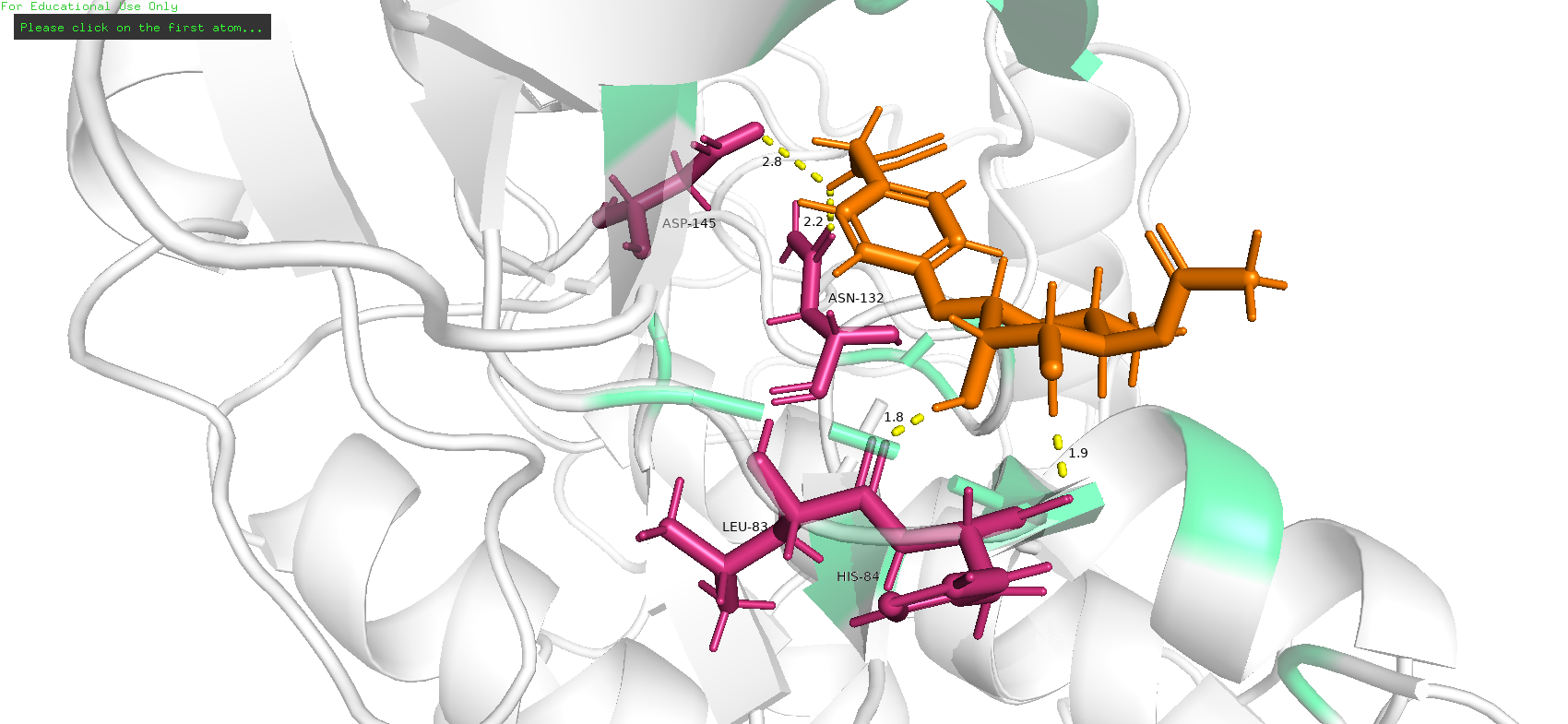


Figure S10: Complex of 4(4’-o-acetyl-alpha-l-rhamnosyloxy) Benzyl isothiocyanate (4) with CDK-2 protein showing polar contacts


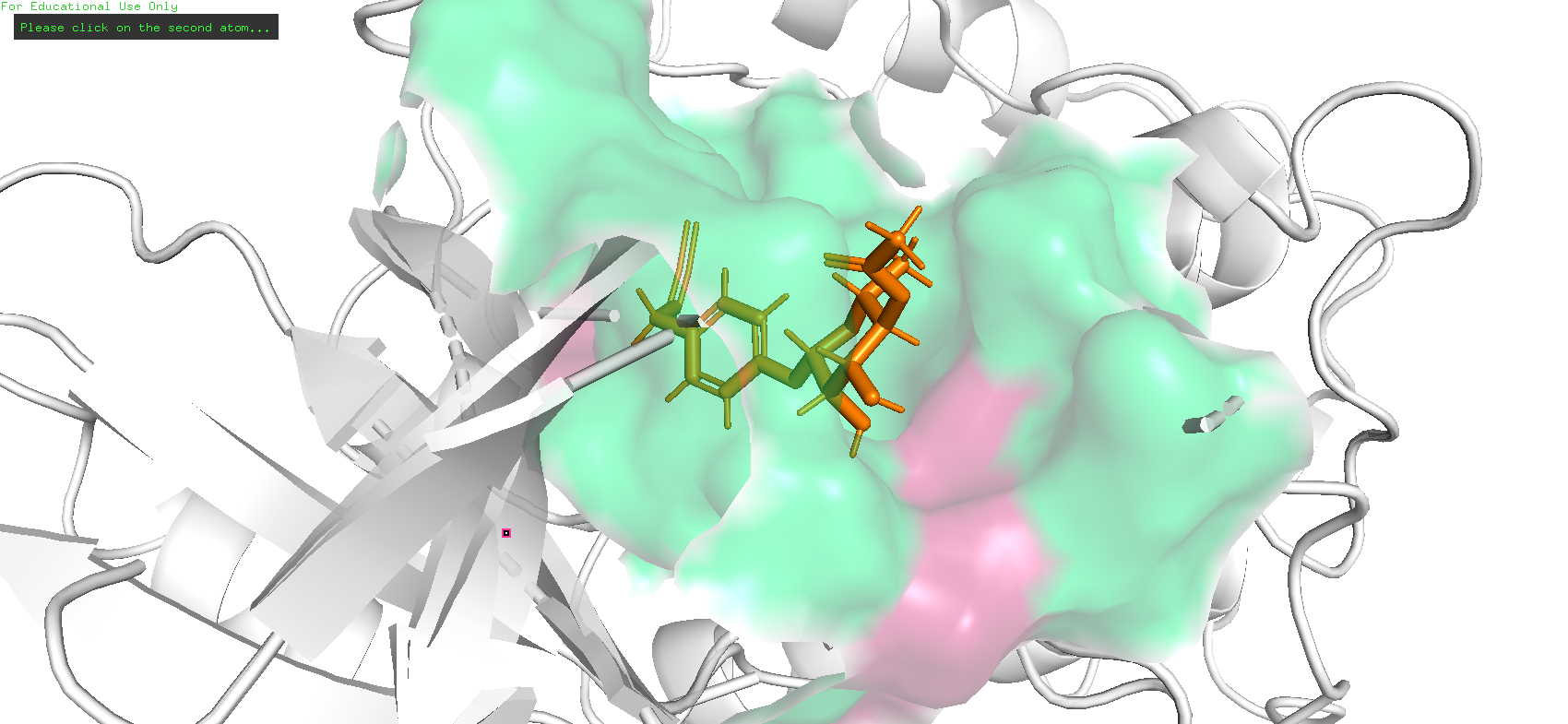


Figure S11: Complex of 4(4’-o-acetyl-alpha-l-rhamnosyloxy) Benzyl isothiocyanate (4) with CDK-2 protein showing non-polar contacts


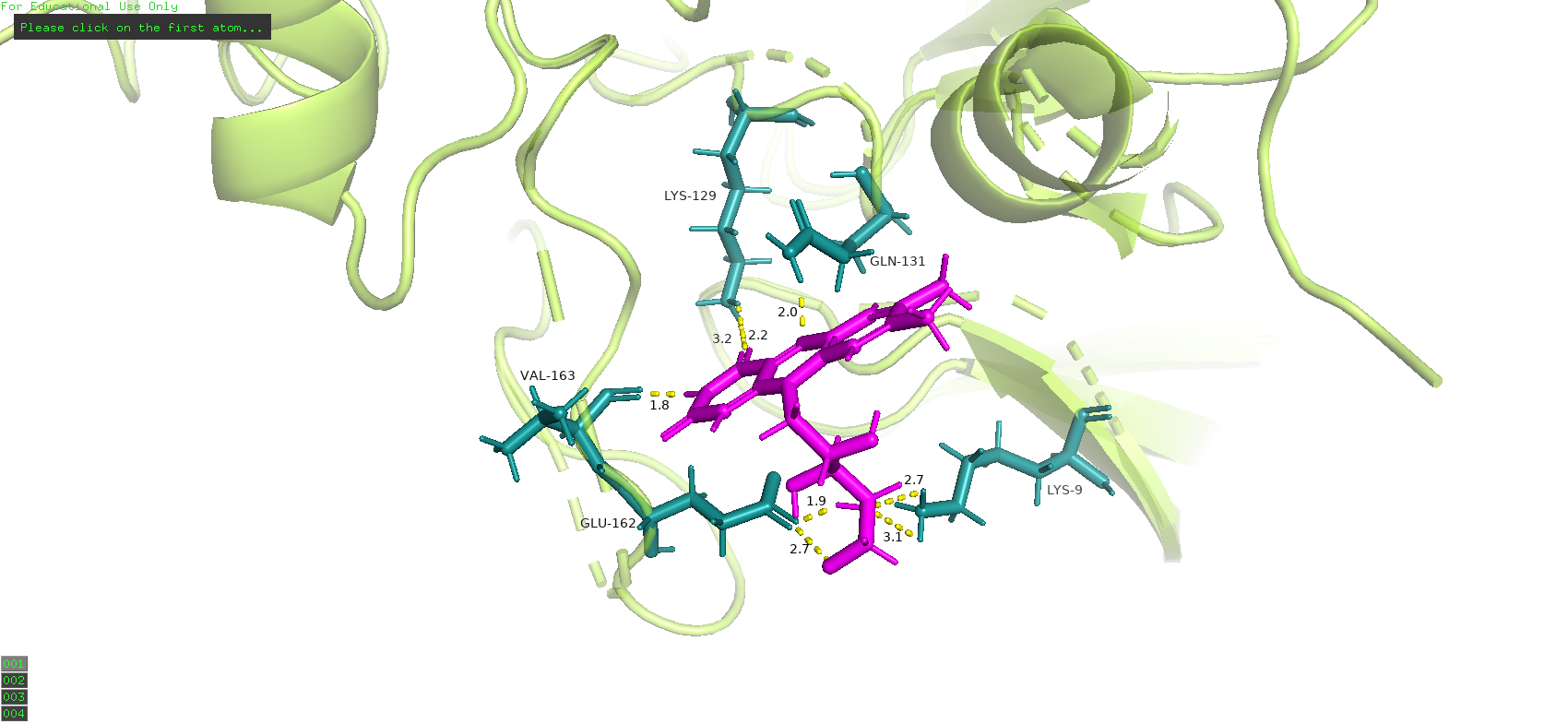


Figure S12: Complex of vitamin B2 (5) with CDK-2 protein showing polar contacts


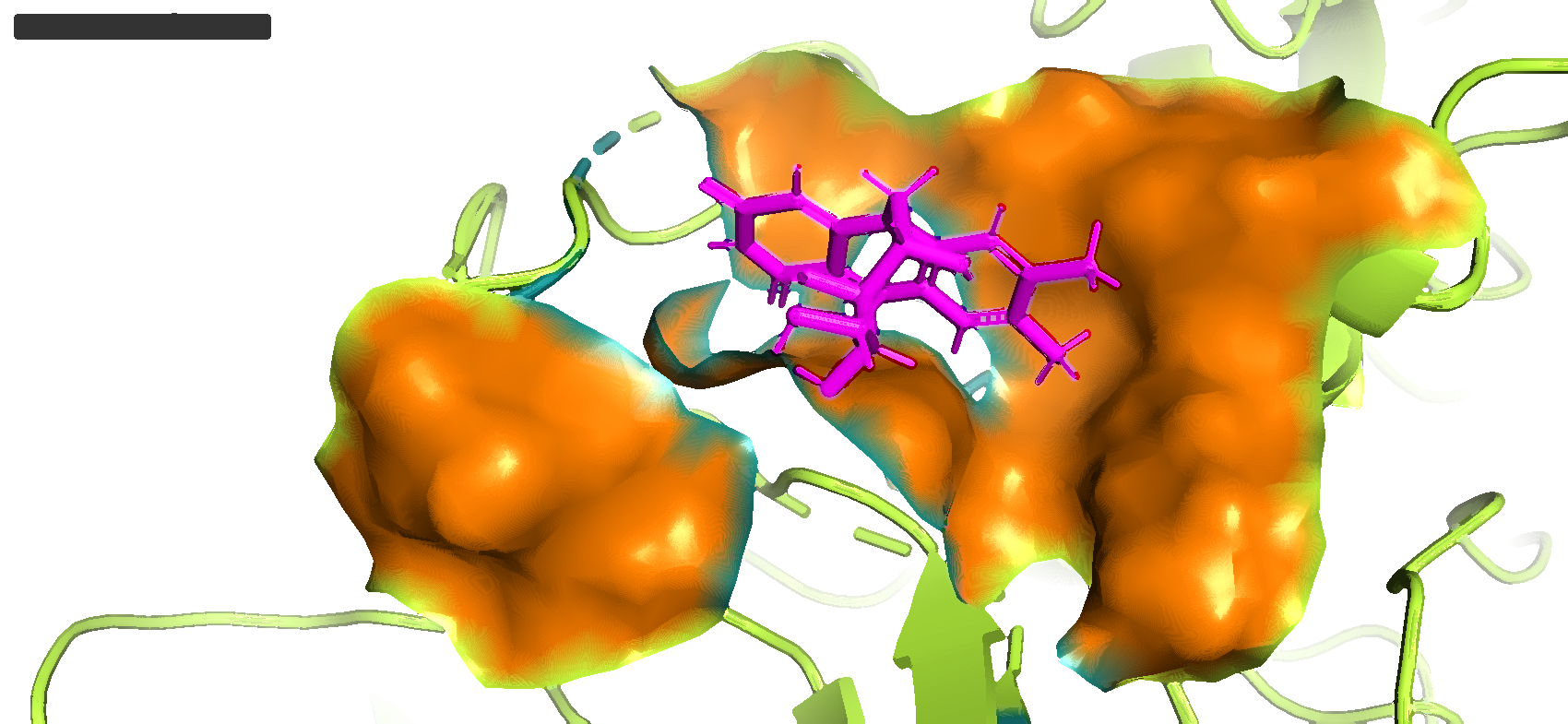


Figure S13: Complex of vitamin B2 (5) with CDK-2 protein showing non-polar contacts


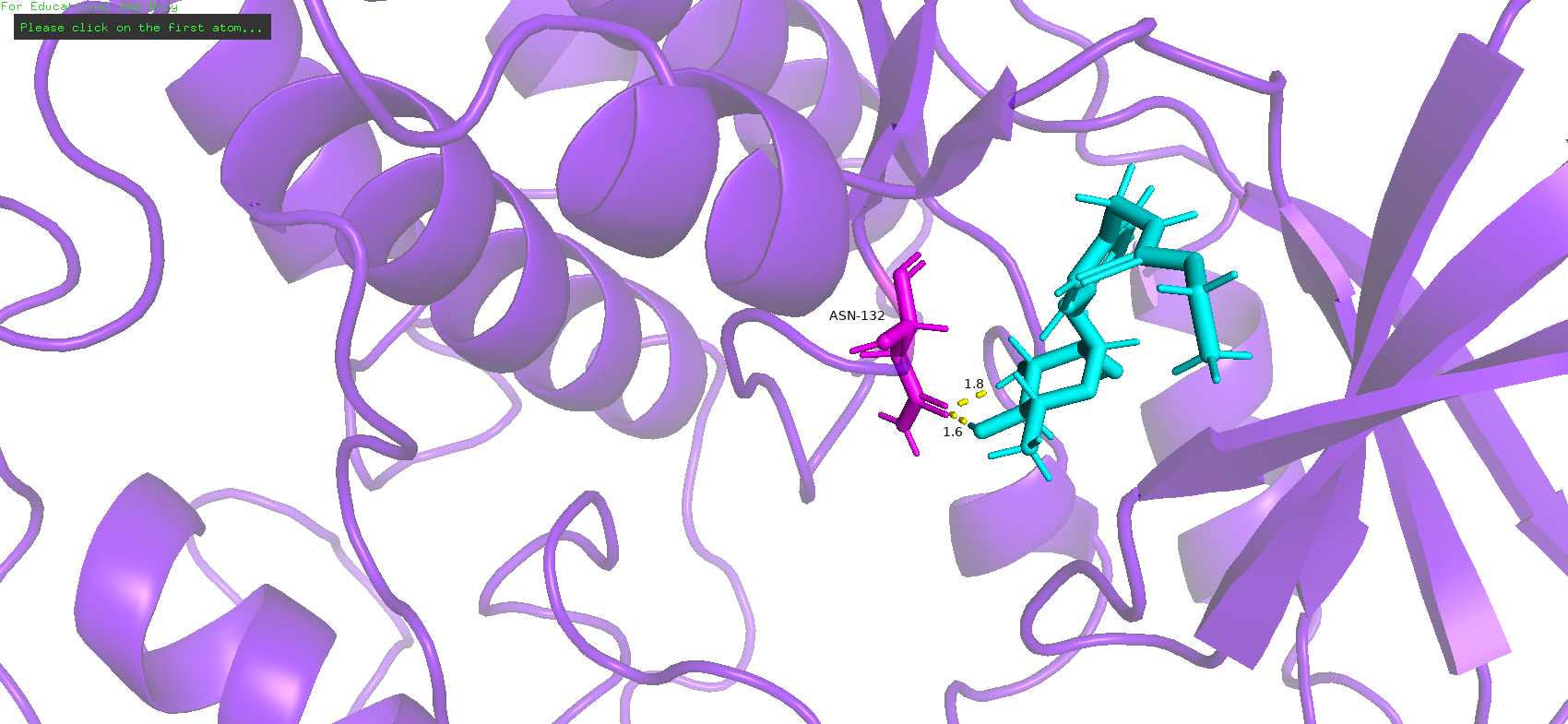


Figure S14: Complex of niazimicin (6) with CDK-2 protein showing polar contacts


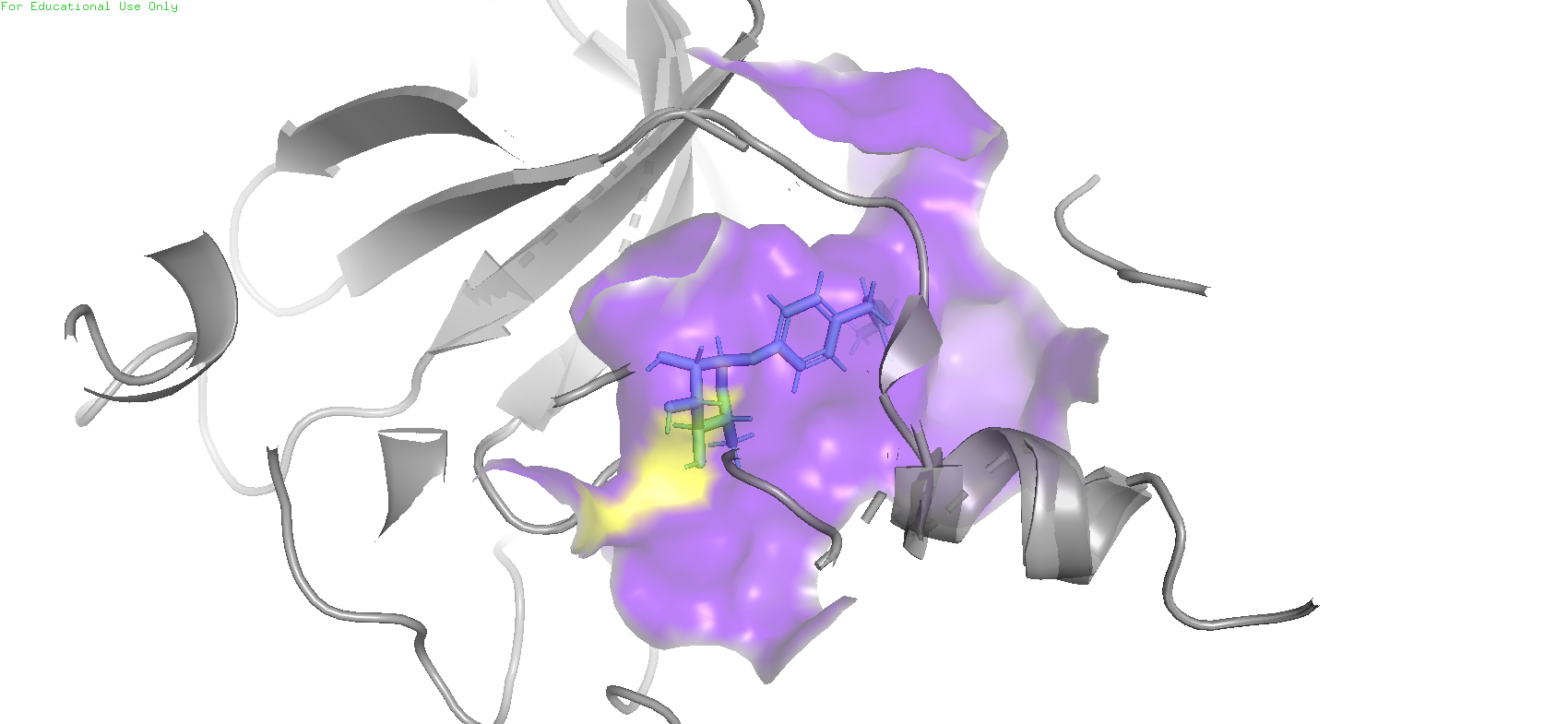


Figure S15: Complex of niazimicin (6) with CDK-2 protein showing non-polar contacts


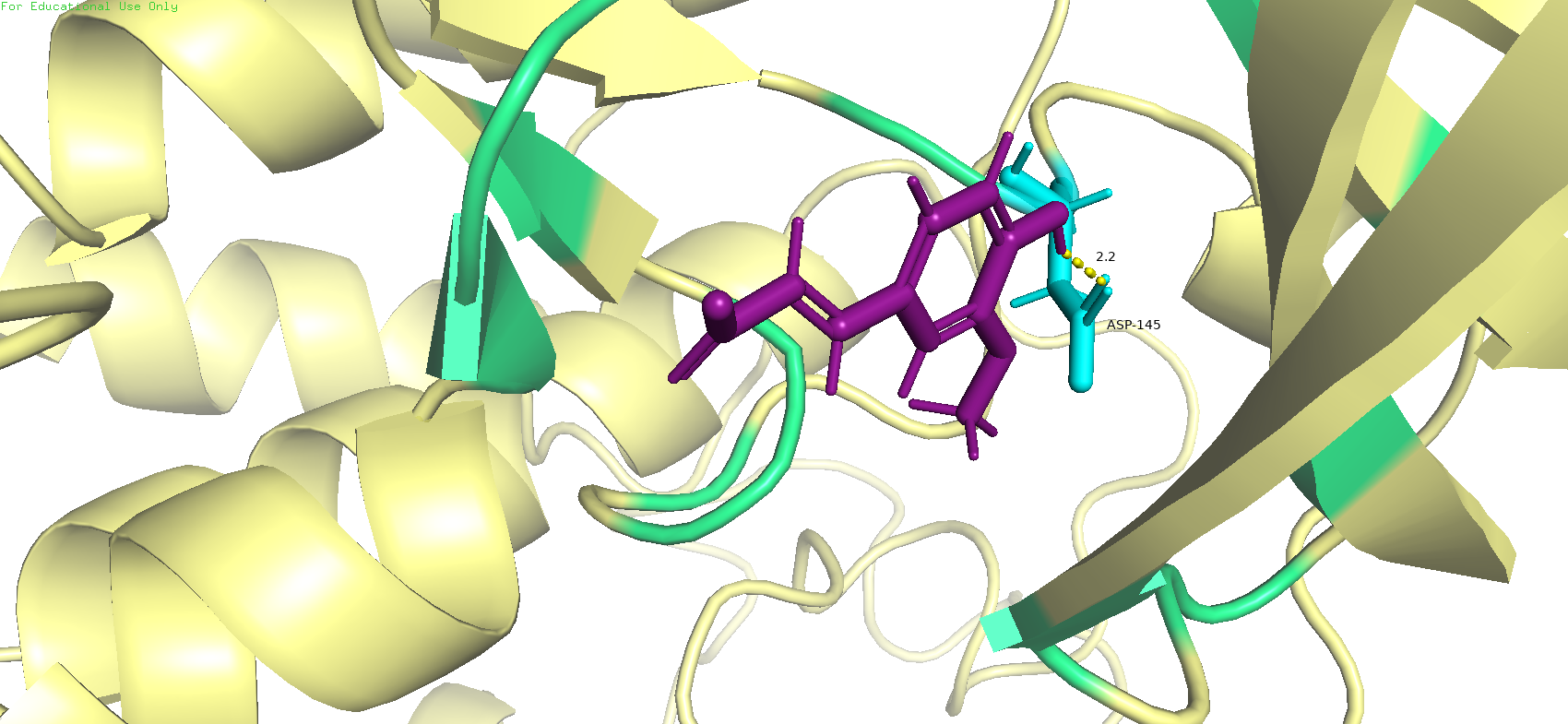


Figure S16: Complex of ferulic acid (7) with CDK-2 protein showing polar contacts


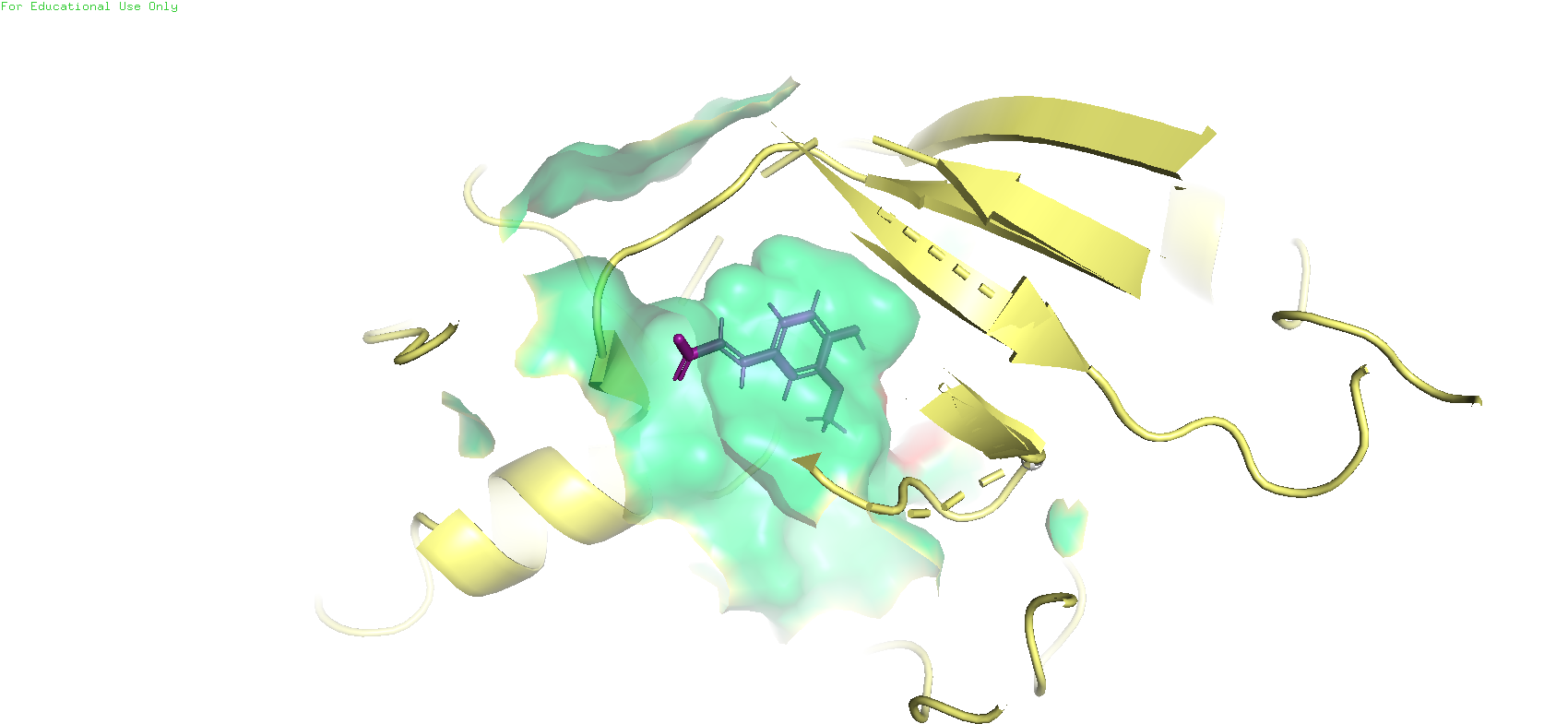


Figure S17: Complex of ferulic acid (7) with CDK-2 protein showing non-polar contacts


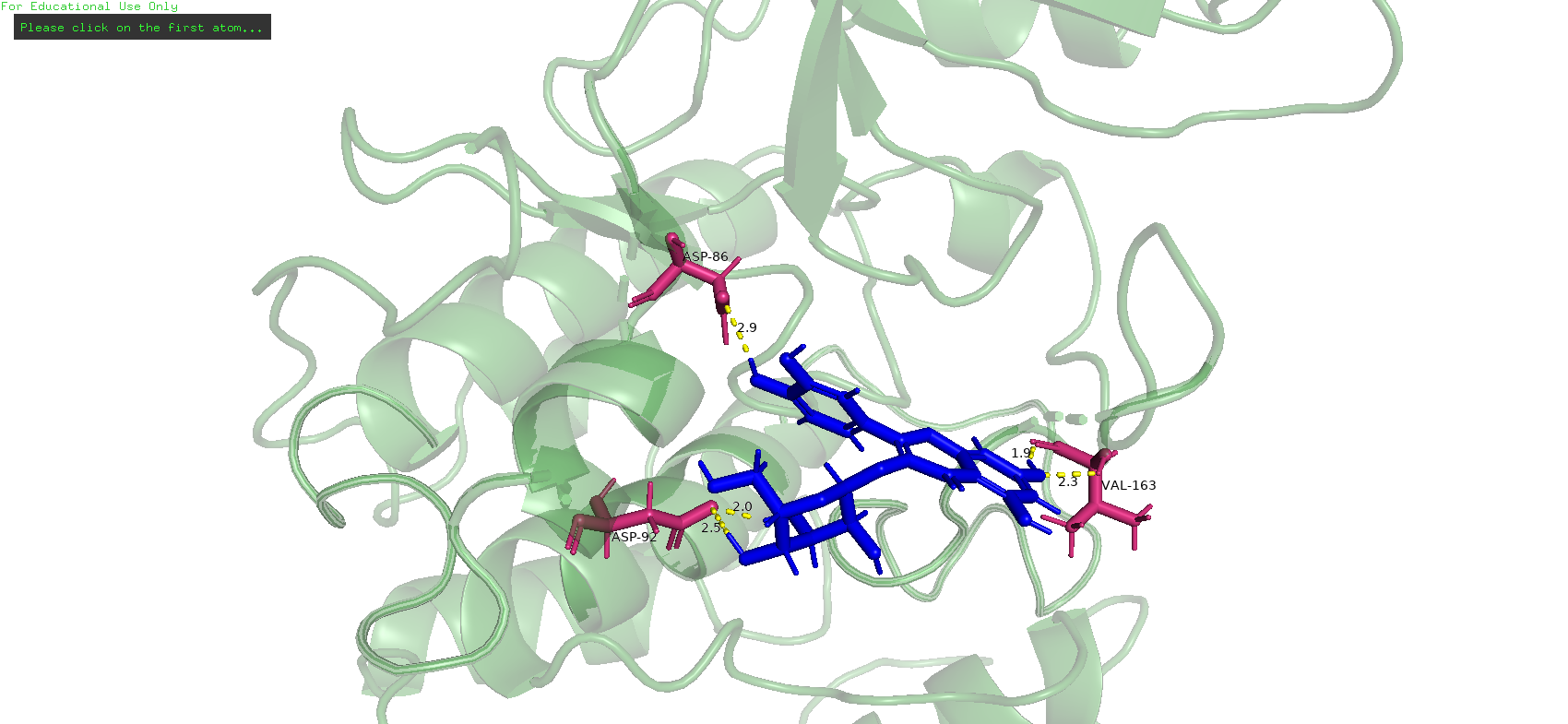


Figure S18: Complex of isoquercetin (8) with CDK-2 protein showing polar contacts


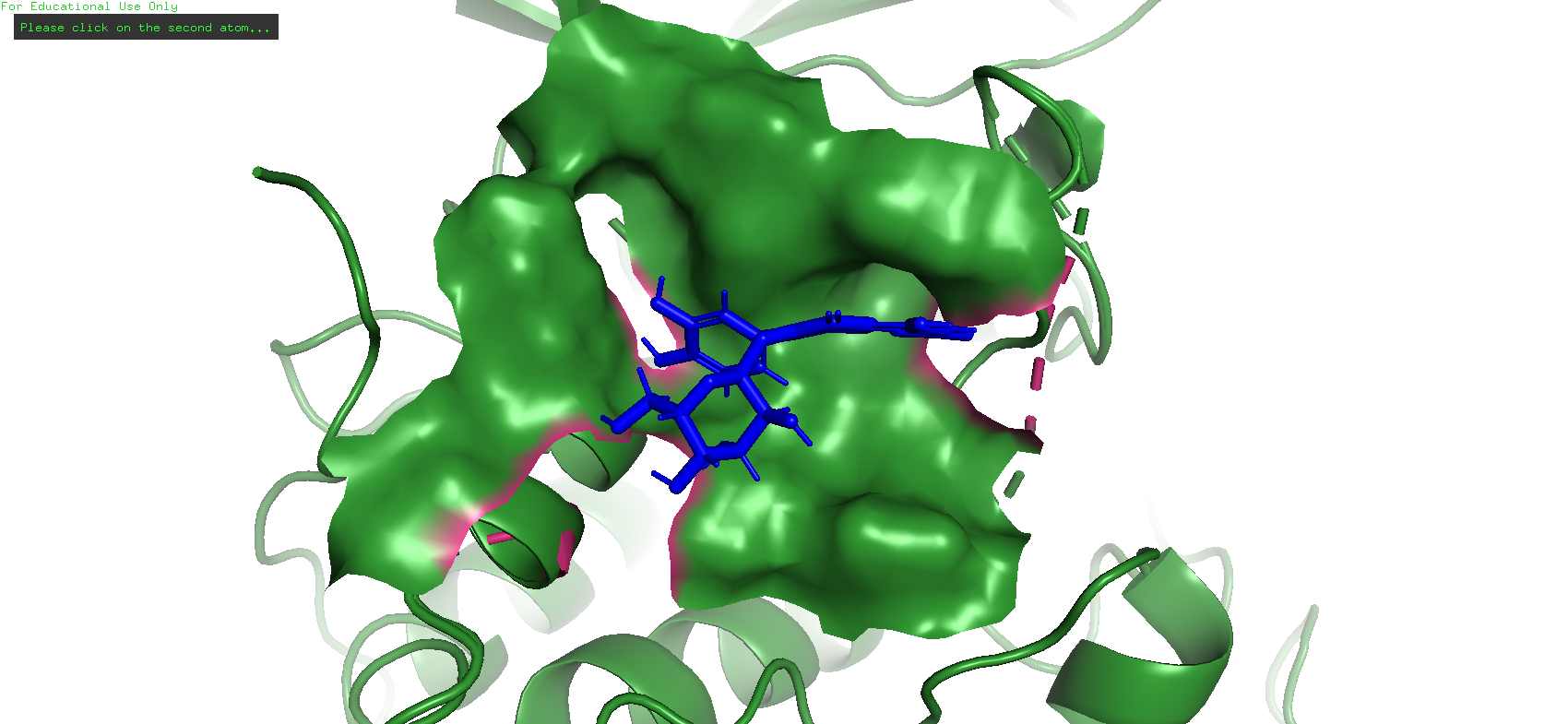


Figure S19: Complex of isoquercetin (8) with CDK-2 protein showing non-polar contacts


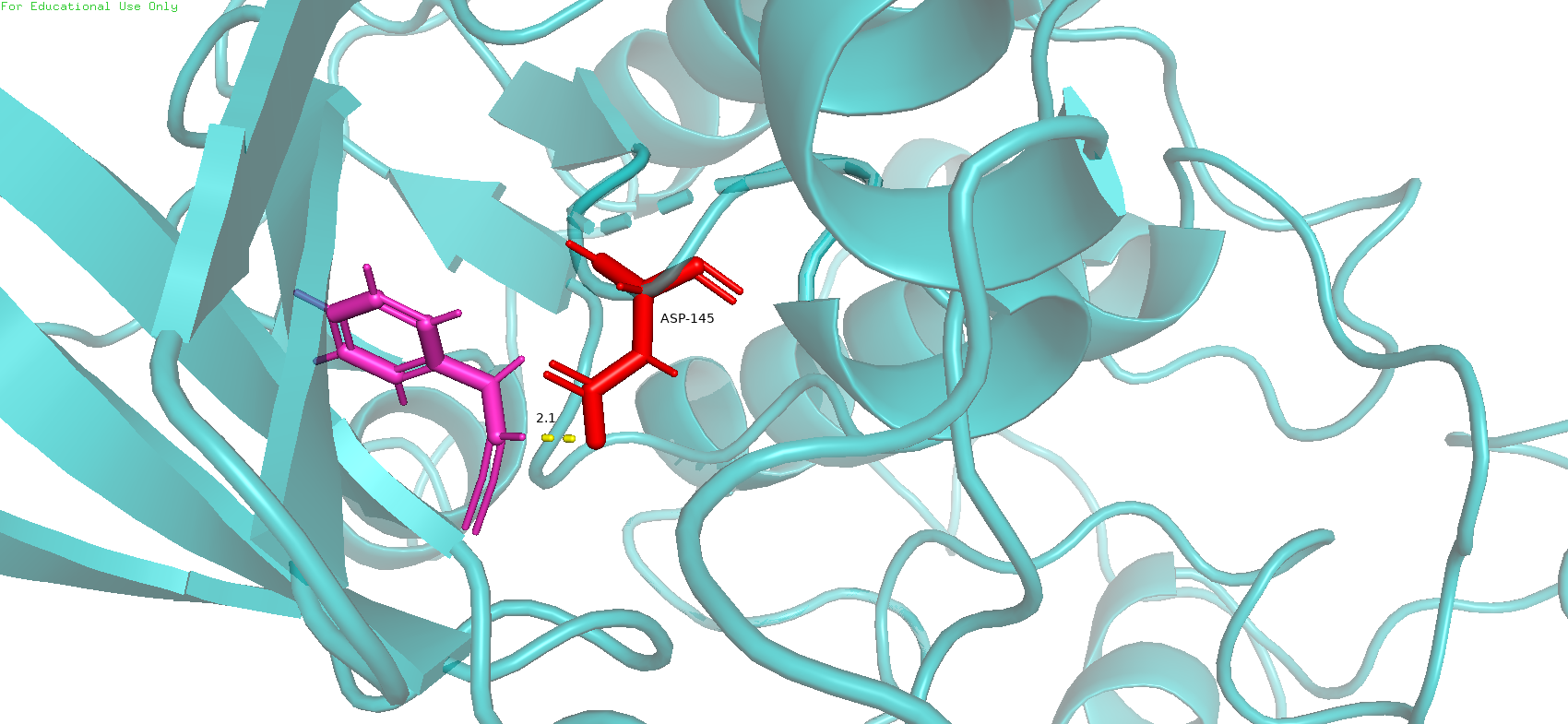


Figure S20: Complex of benzyl-isothiocyanate (9) with CDK-2 protein showing polar contacts


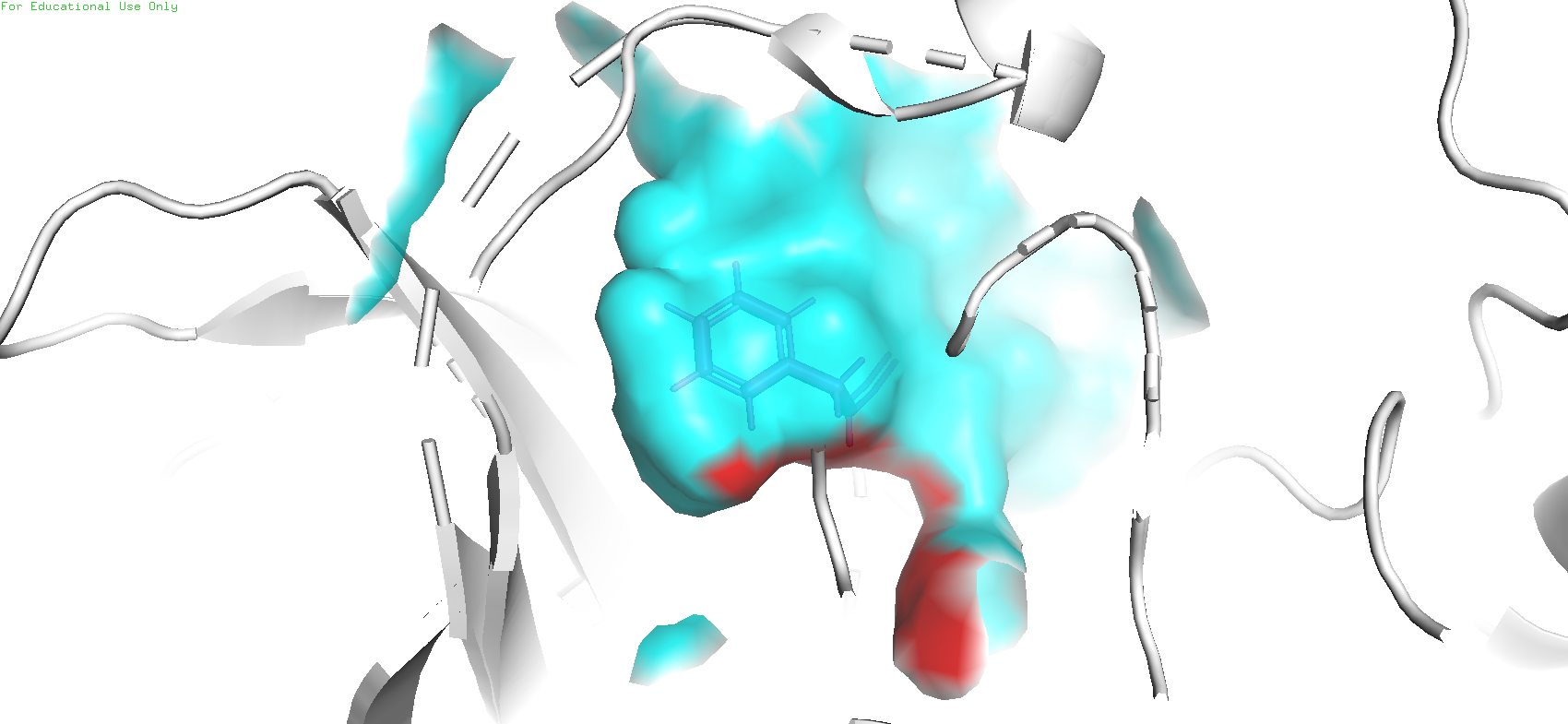


Figure S21: Complex of benzyl-isothiocyanate (9) with CDK-2 protein showing non-polar contacts


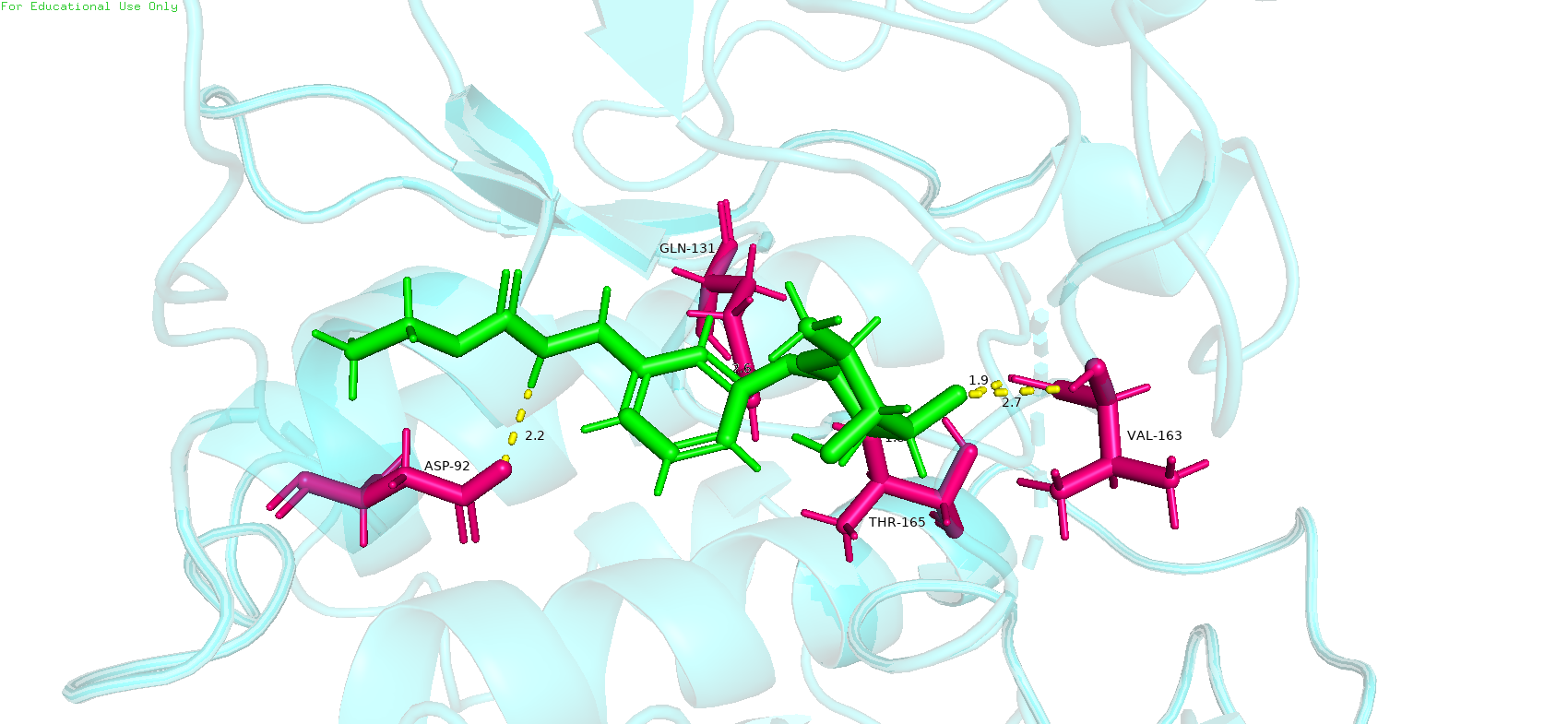


Figure S22: Complex of O-ethyl-4-(alpha-l-rhamnosyloxy) benzyl carbamate (10) with CDK-2 protein showing polar contacts


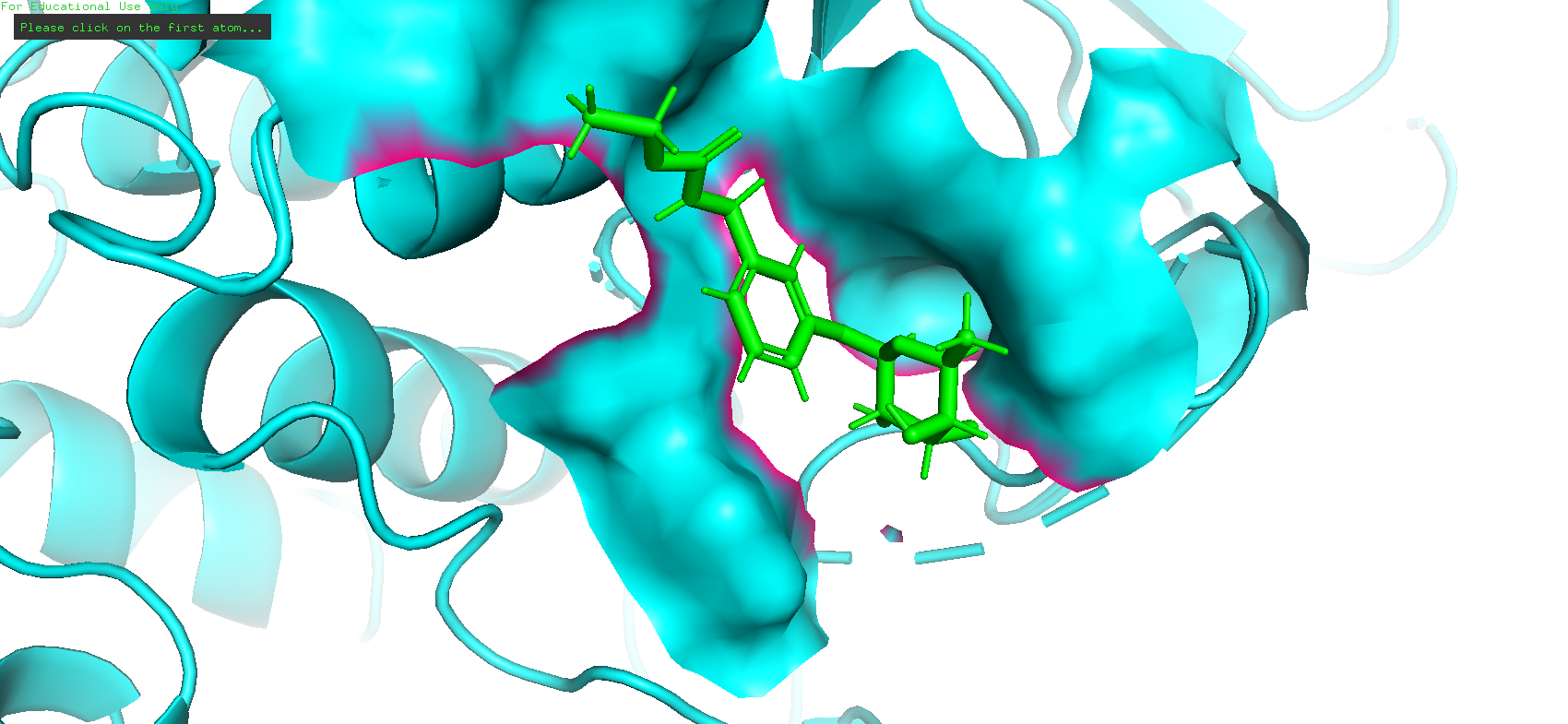


Figure S23: Complex of O-ethyl-4-(alpha-l-rhamnosyloxy) benzyl carbamate (10) with CDK-2 protein showing non-polar contacts


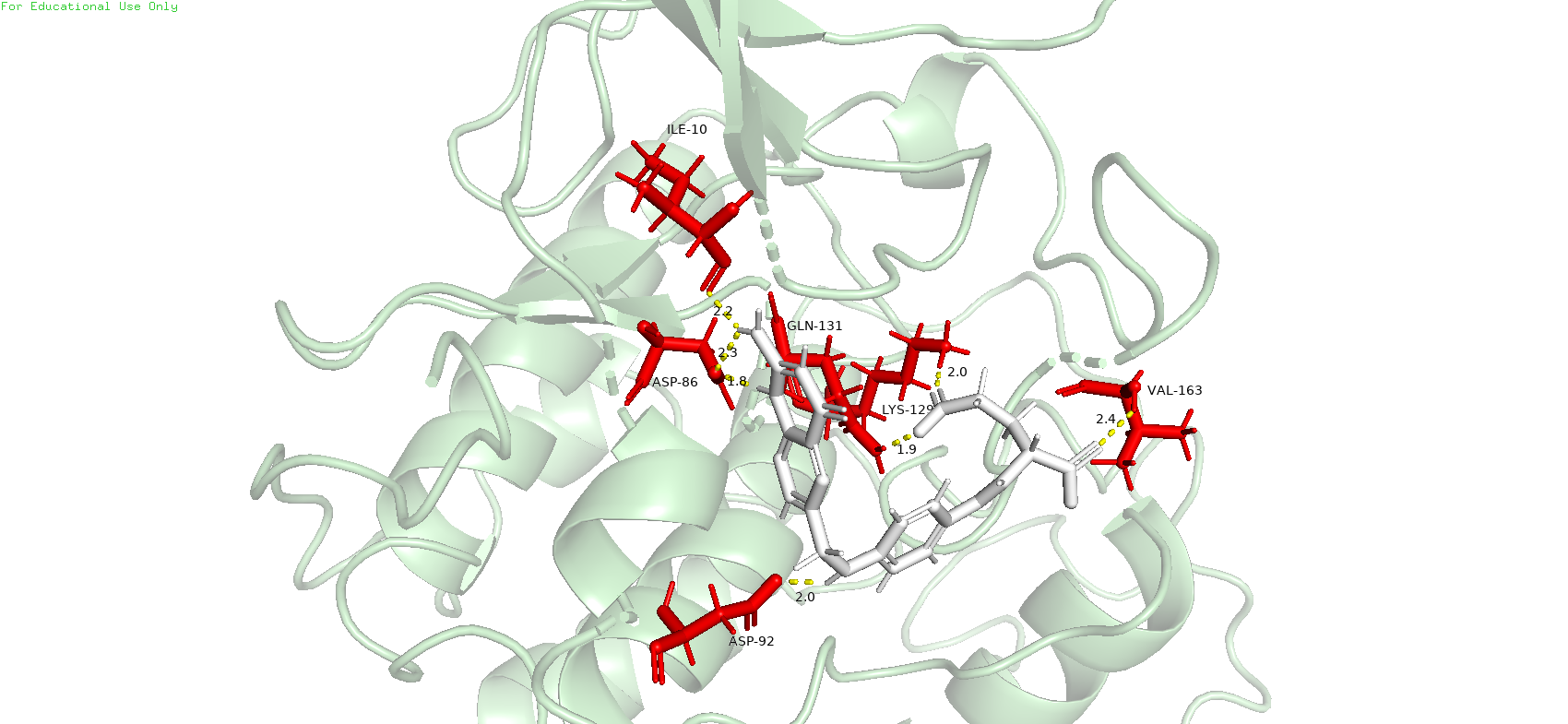


Figure S24: Complex of folic acid (11) with CDK-2 protein showing polar contacts


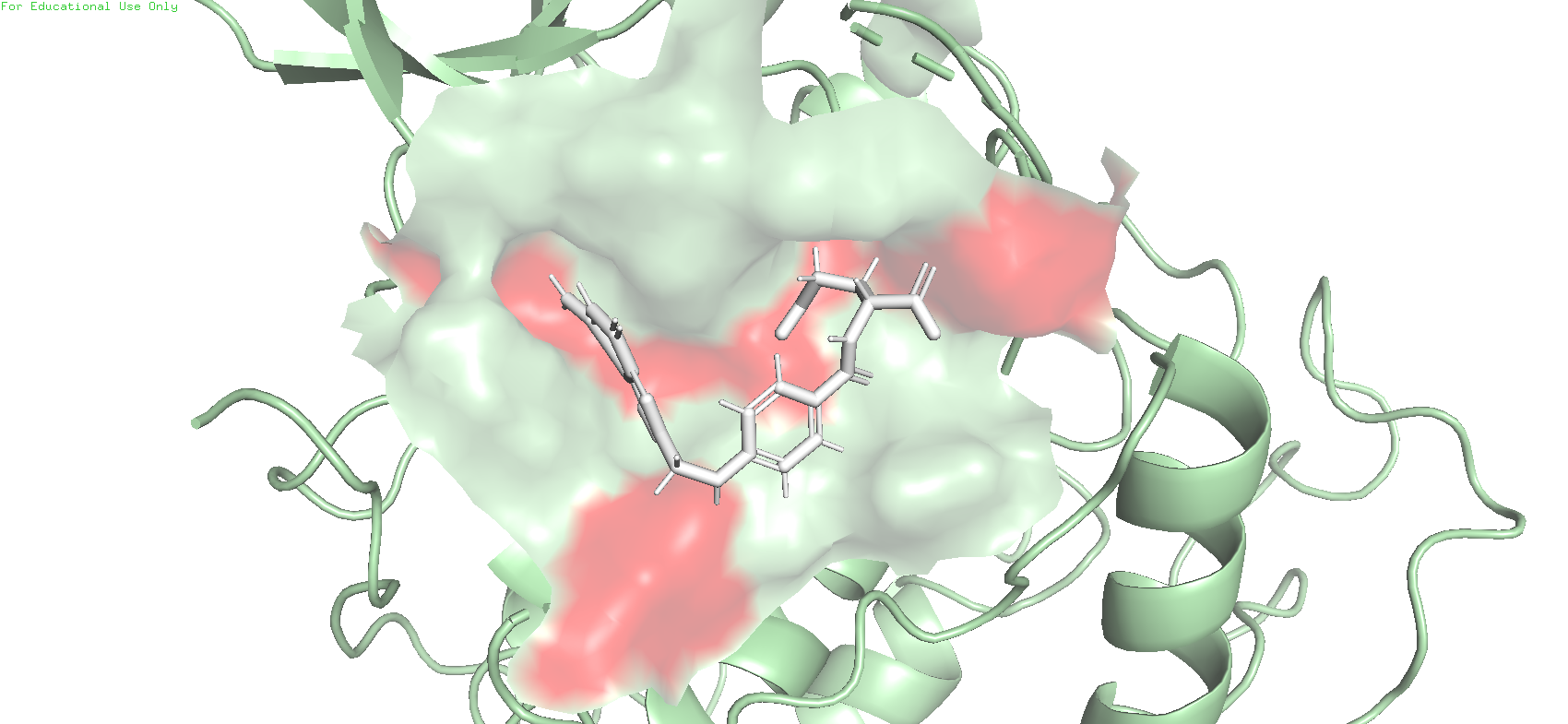


Figure S25: Complex of folic acid (11) with CDK-2 protein showing non-polar contacts


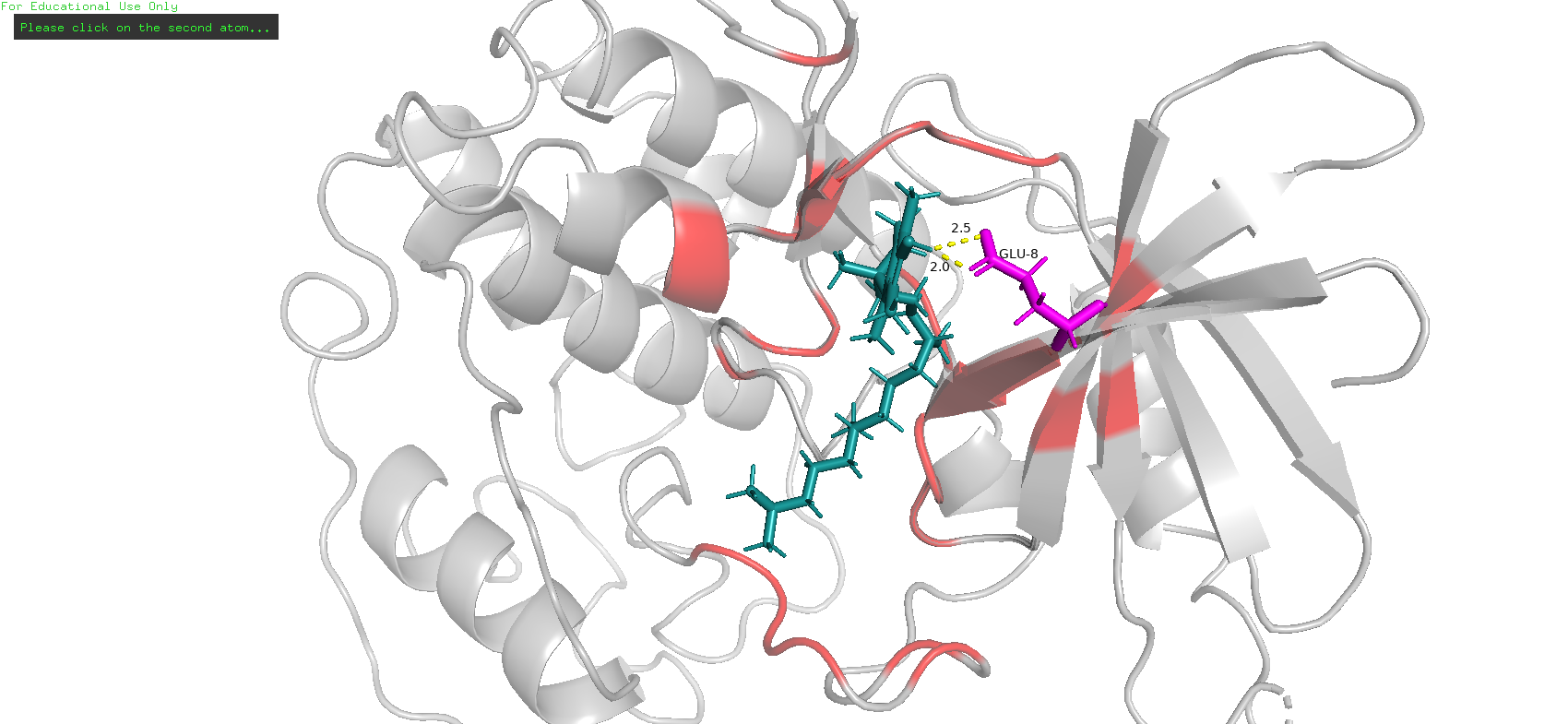


Figure S26: Complex of dl-alpha tocopherol (12) with CDK-2 protein showing polar contacts


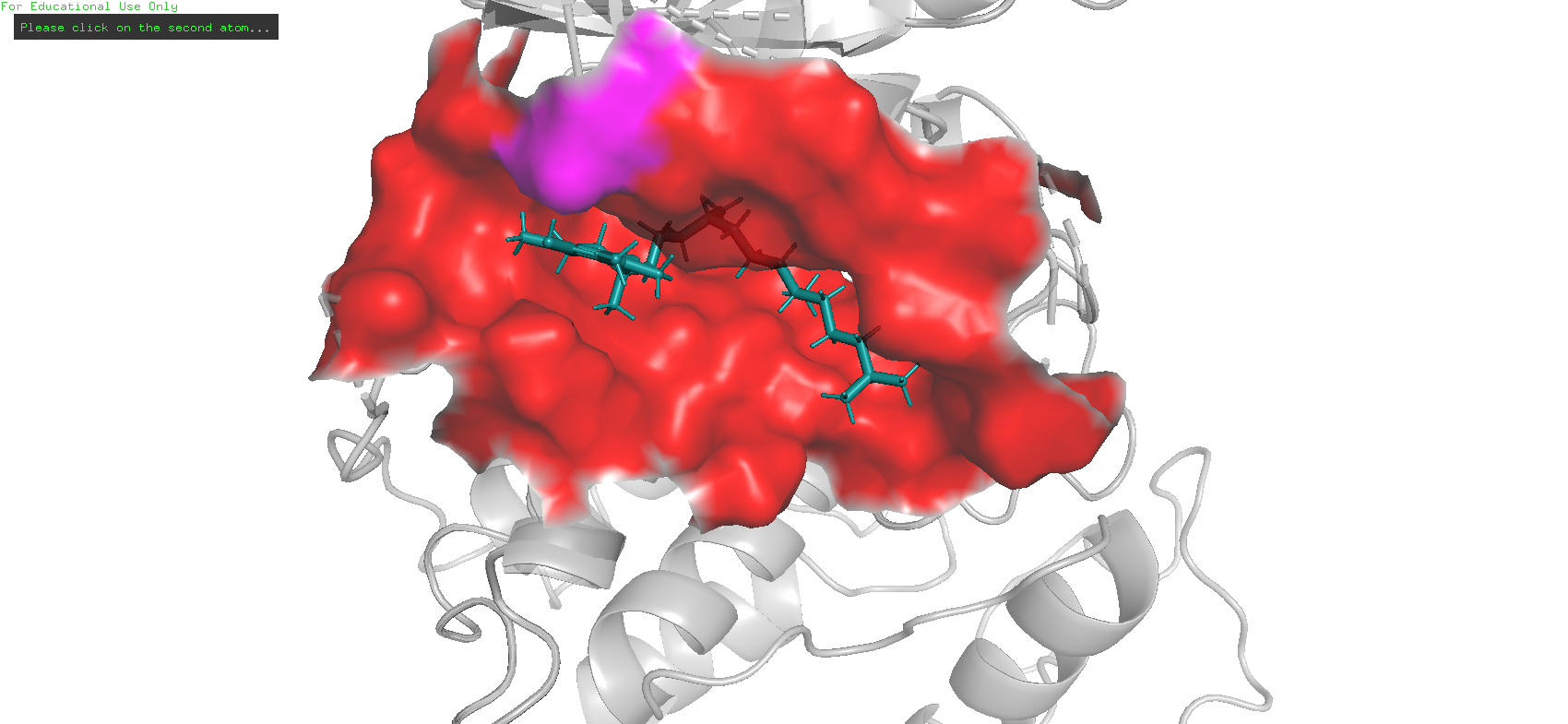


Figure S27: Complex of dl-alpha tocopherol (12) with CDK-2 protein showing non-polar contacts


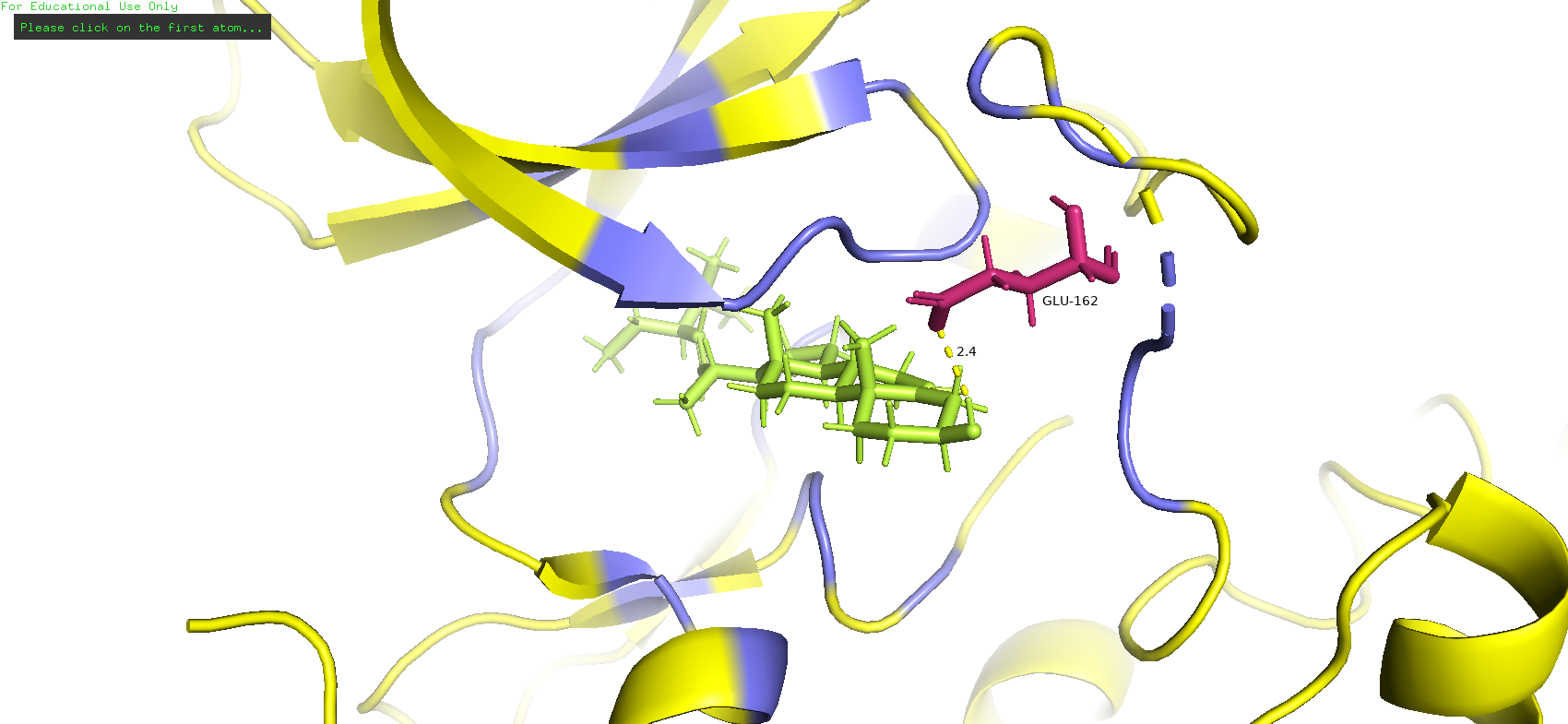


Figure S28: Complex of stigmasterol (13) with CDK-2 protein showing polar contacts


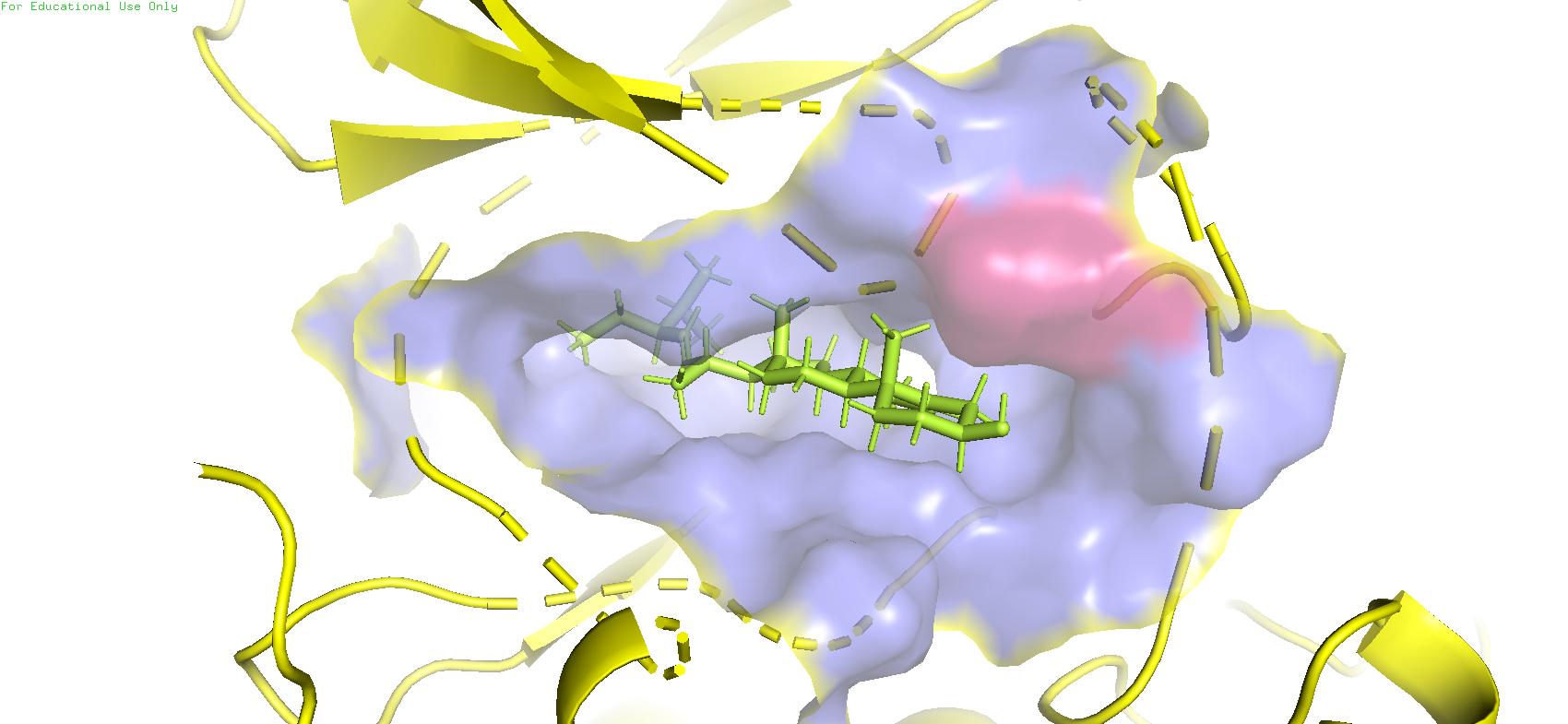


Figure S29: Complex of stigmasterol (13) with CDK-2 protein showing non-polar contacts
